# Determination of the Protein and Amino Acid Content of Fruit, Vegetables and Starchy Roots for Use in Inherited Metabolic Disorders

**DOI:** 10.3390/nu16172812

**Published:** 2024-08-23

**Authors:** Fiona Boyle, Gary Lynch, Clare M. Reynolds, Adam Green, Gemma Parr, Caoimhe Howard, Ina Knerr, Jane Rice

**Affiliations:** 1 Department of Nutrition and Dietetics, National Centre for Inherited Metabolic Disorders, Children’s Health Ireland, Temple Street, D01 XD99 Dublin, Ireland; 2 School of Public Health, Physiotherapy and Sports Science, University College Dublin, Belfield, D04 V2P1 Dublin, Ireland; 3 ALS Laboratories (UK) Limited, ALS Food and Pharmaceutical, Medcalfe Way, Bridge St., Chatteris PE16 6QZ, UK; 4 National Centre for Inherited Metabolic Disorders, Children’s Health Ireland, Temple Street, D01 XD99 Dublin, Ireland

**Keywords:** amino acids, fruits, glutaric aciduria type 1, homocystinuria, inherited metabolic disorders, maple syrup urine disease, methionine, leucine, lysine, phenylalanine, phenylketonuria, protein, starchy roots, tyrosinaemia, tyrosine, urea cycle disorders, vegetables

## Abstract

Amino acid (AA)-related inherited metabolic disorders (IMDs) and urea cycle disorders (UCDs) require strict dietary management including foods low in protein such as fruits, vegetables and starchy roots. Despite this recommendation, there are limited data on the AA content of many of these foods. The aim of this study is to describe an analysis of the protein and AA content of a range of fruits, vegetables and starchy roots, specifically focusing on amino acids (AAs) relevant to AA-related IMDs such as phenylalanine (Phe), methionine (Met), leucine (Leu), lysine (Lys) and tyrosine (Tyr). AA analysis was performed using high-performance liquid chromatography (HPLC) on 165 food samples. Protein analysis was also carried out using the Dumas method. Foods were classified as either ‘Fruits’, ‘Dried fruits’, ‘Cruciferous vegetables’, ‘Legumes’, ‘Other vegetables’ or ‘Starchy roots’. ‘Dried fruits’ and ‘Legumes’ had the highest median values of protein, while ‘Fruits’ and ‘Cruciferous vegetables’ contained the lowest median results. ‘Legumes’ contained the highest and ‘Fruits’ had the lowest median values for all five AAs. Variations were seen in AA content for individual foods. The results presented in this study provide useful data on the protein and AA content of fruits, vegetables and starchy roots which can be used in clinical practice. This further expansion of the current literature will help to improve diet quality and metabolic control among individuals with AA-related IMDs and UCDs.

## 1. Introduction

Inherited metabolic disorders (IMDs) are a heterogeneous class of genetic disorders that result in impairment of metabolic pathways [1]. Defects in these pathways can cause a build-up of metabolites which can become toxic and a relative deficiency of other essential substances [2]. Phenylketonuria (PKU), maple syrup urine disease (MSUD), homocystinuria (HCU), glutaric aciduria type 1 (GA1) and tyrosinaemia I, II and III are types of IMDs that are caused by deficiencies of enzymes involved in the metabolism of amino acids (AAs) [3]. Urea cycle disorders (UCDs) are a group of IMDs caused by a deficiency of the enzymes required to convert toxic ammonia into non-toxic urea for excretion in the kidney and in the biosynthesis of arginine and citrulline [3]. Advancements in screening, diagnosis and clinical management have improved clinical outcomes and life expectancy among patients with these IMDs [4]. These rare disorders, however, require lifelong treatment including strict restriction of natural protein (and hence specific AAs), consumption of a condition-specific synthetic protein substitute and inclusion of foods naturally low in protein and specially manufactured low-protein foods [2]. Without appropriate treatment, IMDs can affect multiple organs and systems of the body leading to neurological issues, severe learning and physical disabilities, illness and mortality [2,3,5]. 

In AA-related IMDs and UCDs, although restriction of specific AAs or proteins in the diet is necessary to avoid toxicity, each patient will have a minimum physiological requirement for growth, development and maintenance of lean body mass [6]. Each individual will also have their own individual tolerance of the specific amino acid (AA) or protein depending on the residual activity of the affected enzyme [2,4]. Internationally, families in many metabolic clinics are educated on counting natural protein intake instead of individual amino acid intake as a simplified approach to the dietary management of AA-related IMDs [3]. In the Republic of Ireland, protein counting is taught as a 1 g protein exchange system as a practical way of monitoring protein and AA intake. Patients are allocated a daily natural protein allowance to be spread out over the course of the day. This recommended allowance will change over the lifetime depending on the individual’s plasma or dried blood AA results, with the aim of maintaining AA levels within a therapeutic target range [2,3,7]. 

While the simplified diet approach is a practical method to monitor protein as a proxy of AA intake, it does not accurately reflect the AA content of all foods. The contribution of AAs to the total protein content of foods differs between plant foods and animal foods. For example, it is generally accepted that animal milk and cereal protein sources contain approximately 5% phenylalanine (Phe) (50 milligrams (mg) of Phe per 1 g of protein), while fruit and vegetable protein sources contain approximately 3–4% Phe (30–40 mg of Phe per 1 g of protein) [8,9]. Similar differences have also been observed regarding the contribution of other AAs to the total protein content of plant foods and animal foods [10,11,12]. The simplified diet approach, however, allows greater flexibility with the diet and encourages a more varied diet and healthy food choices when compared with counting intake of AAs [13]. 

Many fruits, vegetables and starchy roots are naturally low in protein and hence low in AAs [3,14]. In the simplified diet approach, certain fruits, vegetables and starchy roots that contain lower amounts of protein and AAs are permitted for consumption in the diet without measuring or counting [3,13]. Other fruits and vegetables need to be incorporated into the natural protein allowance due to their relatively higher protein and AA content [3,14,15]. The ‘Complete European guidelines on PKU diagnosis and treatment’, for example, recommend that fruits and vegetables with a Phe content of less than 75 mg/100 g of food (excluding potatoes) can be permitted without counting into the natural protein allowance for those with PKU [14]. This recommendation was based on a number of studies that found that including these foods did not adversely affect blood Phe levels [16,17,18,19,20,21,22,23]. Of note, fruits and vegetables allowed freely or counted as part of the natural protein allowance vary between IMDs and also between metabolic clinics [13,23].

Due to the high cost of testing for AA content, data on the AA content of foods are limited. In 2006, the National Society for Phenylketonuria (NSPKU) in the United Kingdom (UK) commissioned the Phe analysis of 172 foods, predominantly focusing on fruits and vegetables [24]. This was the first UK publication of dietary Phe analysis since ‘McCance and Widdowson’s Composition of Foods’ in 1980 which contained analysis of a limited number of fruits and vegetables [12]. A recent publication by Ford et al. detailed the AA analysis of 73 plant foods including many novel plant foods [9]. The analysis included information on the Phe, leucine (Leu), methionine (Met), tyrosine (Tyr) and lysine (Lys) content of these foods. These publications have provided invaluable information on the AA content of a wide variety of plant foods for which data were limited for use in AA-related IMDs. 

The aim of this study is to describe a further analysis of the protein and AA content of fruits, vegetables and starchy roots, specifically focusing on AAs most relevant to IMDs (Phe, Met, Leu, Lys and Tyr). As part of this study, we aim to expand on the existing literature and databases on the protein and AA content of fruits, vegetables and starchy roots, including many foods for which there is limited or no data. A further aim of this study is to expand on the number of fruits, vegetables and starchy roots that are allowed freely in AA-related IMDs and UCDs, provided that their AA and protein content are sufficiently low using new data from this study.

## 2. Materials and Methods

### 2.1. Preparation of Food Samples

Fruits, vegetables and starchy roots were selected for analysis of protein and AA content by two experienced metabolic dietitians in consultation with their colleagues both in the National Centre for Inherited Metabolic Disorders (NCIMD), Children’s Health Ireland (CHI), Dublin, Ireland, families attending NCIMD and colleagues internationally. Fruits, vegetables and starchy roots that had either limited or no AA data were included. Foods were also selected for analysis where varied approaches existed internationally, on inclusion towards the natural protein allowance. 

Foods for the analysis were purchased from Irish supermarkets (Tesco, Dunnes Stores; Dublin, Ireland) and from local fresh produce stores. Where possible, fresh fruits, vegetables and starchy roots were chosen for analysis. In some cases, fruits and vegetables were tinned (e.g., palm seeds, lychees); dried (e.g., cranberries, goji berries); jarred (e.g., cornichons, grilled artichokes) or frozen (e.g., brussel sprouts, spinach). 

Foods were prepared by metabolic dietitians and student dietitians in NCIMD as per instructions detailed in Supplement Table S1. A 200 g sample of each food was portioned into an airtight container and refrigerated. Prior to portioning, the typical portion size (average/adult and small/child) of each food was estimated, measured and recorded. The samples were then transported under ice blocks overnight to the ALS Life Sciences laboratory in Chatteris, Cambridgeshire, United Kingdom for protein and AA analysis. ALS Life Sciences is accredited to ISO standard 17025:2017 (https://www.iasonline.org/services/testing-laboratories/, accessed on 21 June 2024) by United Kingdom Accreditation Service (UKAS) for the analysis of Nitrogen/Protein by Dumas and Amino Acid Determination by high-performance liquid chromatography (HPLC). 

### 2.2. Laboratory Analysis

Before analysis, dry food samples were ground and wet food samples were homogenised to provide a representative sample for analysis. Prepared samples were then stored in airtight containers. Dry foods were stored at room temperature and wet foods were stored at 2–8 °C. The food samples were then treated with 6 M hydrochloric acid in sealed bottles at 115 ± 5 °C for 7 h to hydrolyse the protein chains into their component AAs and peptides. To analyse cystine and methionine, the food samples were oxidised with performic acid at 2–8 °C for 16 h before acid hydrolysis. The hydrolysate was then diluted and filtered. An aliquot of the sample was adjusted to pH 2.4 and a predetermined amount of norleucine was added as an internal standard before dilution. Analysis of samples with free AAs were extracted with hydrochloric acid before dilution and the addition of an internal standard. Once extracted, the AAs were derivatised with 6-aminoquinolyl-N-hydroxysuccinimidyl carbamate (AccQ-Fluor). The AA content was quantified by gradient HPLC with fluorescence detection and a chromatography data handling system (Chromeleon^TM^) (HPLC operational requirements including HPLC setup and chromatographical interpretation conducted as per ALS method AM/V/206 issue 23). This method allowed quantitative detection of individual AA concentrations to a limit of quantification of 5 mg/100 g of food. The Phe, Met, Leu, Lys and Tyr content of each food sample in mg/100 g was reported. Protein analysis was carried out using the Dumas method whereby protein content was determined from the nitrogen content using an appropriate conversion factor. This method allowed quantitative detection of individual protein levels to a limit of quantification of 0.1 g/100 g of food. One single measurement of protein, Phe, Met, Leu, Lys and Tyr per 100 g was recorded for each food sample analysed. 

### 2.3. Data Analysis

Following the analysis, the protein, Phe, Met, Leu, Lys and Tyr content of the selected foods were evaluated. The foods were arranged into the following categories: ‘Fruits’, ‘Dried fruits’, ‘Cruciferous vegetables’, ‘Legumes’, ‘Other vegetables’ and ‘Starchy roots’. When distinguishing our food samples, ‘Fruits’ were defined as the edible part of the plant which contains the seeds and pulpy surrounding tissue, while ‘Vegetables’ were defined as the non-seed-bearing parts of the plant [25]. ‘Starchy roots’ were defined as plants which store edible starch material in subterranean stems, roots, rhizomes, corms and tubers [26]. In the ‘Vegetable’ subgroups, vegetables belonging to the Brassicaceae family were classified as ‘Cruciferous vegetables’ [27]; vegetables from the Fabaceae family were grouped as ‘Legumes’ [28]; and all other vegetables were classed as ‘Other vegetables’. 

Data analysis was performed using the IBM Statistical Package for the Social Sciences (SPSS) version 27. The mean, standard deviation (SD), median and range were calculated for the protein content in grams and AA content in mg per 100 g of the edible portion of food, the AA content (mg) per gram of protein and the percentage (%) of AA content per gram of protein for each food category. Spearman’s rank correlation coefficient test was performed to determine the correlation between the protein content and the individual AA content of food samples.

For our analysis, where more than one sample of the same food cooked using the same method was analysed, an average value was used for the pooled analysis. Protein data less than 0.1 g per 100 g of food and AA data less than 5 mg per 100 g of food were excluded from the analysis.

## 3. Results

### 3.1. Protein and Amino Acid Content per 100 g of Food

In total, 165 samples of fruits, vegetables and starchy roots were analysed to determine their protein and AA content. The protein and AA content of 52 ‘Fruits’, 13 ‘Dried fruits’, 24 ‘Cruciferous vegetables’, 31 ‘Other vegetables’, 6 ‘Legumes’ and 39 ‘Starchy roots’ per 100 g of edition portions of all food samples are presented in Table 1. The protein and amino acid content per typical adult and child portion sizes of selected foods are presented in Supplements Tables S2–S7.

Multiple samples of avocado (*n* = 2), sweet potato (orange, baked) (*n* = 3), sweet potato (orange, boiled) (*n* = 3), sweet potato (orange, roasted) (*n* = 3) and sweet potato (white, boiled) (*n* = 2) were analysed. Although data were similar between each pair of samples for avocado, there were noticeable differences in the protein and AA content between samples of sweet potato. 

### 3.2. Protein and Amino Acid Analysis in Fruits and Vegetables Categories

The mean, SD, median and range of protein and AAs in food categories (*n* = 156) are presented in Table 2. For median values, ‘Legumes’ contained the highest protein per 100 g (2.8 g/100 g) while ‘Fruits’ (0.9 g/100 g) had the lowest. After ‘Legumes’, the highest median values for Phe, Leu, Lys and Tyr were from ‘Other vegetables’. ‘Fruits’ had, overall, the lowest median values for all five AAs of interest in this study. From all the vegetable groups analysed, ‘Cruciferous vegetables’ had the lowest median values for protein, Phe, Met, Leu, Lys and Tyr.

### 3.3. Amino Acid Content (mg) per Gram of Protein

The AA content (mg) per gram of protein of sample foods (*n* =156) is presented in Table 3. The median value of individual AAs ranged from 15 to 50 mg per gram of protein for ‘Fruits’; 8 to 36 mg per gram of protein for ‘Dried fruits’; 14 to 55 mg per gram of protein for ‘Cruciferous vegetables’; 11 to 50 mg per gram of protein for ‘Legumes’, 14 to 50 mg per gram of protein for ‘Other vegetables’ and 20 to 56 mg per gram of protein for ‘Starchy roots’. For individual foods, large variations in AA content per gram of protein were evident, for example, in ‘Cruciferous vegetables, Phe content per g protein, varied from 17 mg for ‘mooli (daikon), white, raw’ to 49 mg for ‘Cabbage, york, boiled’.

### 3.4. Percentage (%) of Amino Acids per Gram of Protein

The % of AAs per gram of protein of sample foods (*n* = 156) is presented in Table 4. The median % of protein provided by individual AAs ranged from 1.5 to 5.0% per gram of protein for ‘Fruits’; 0.8 to 3.6% per gram of protein for ‘Dried fruits’; 1.4 to 5.5% per gram of protein for ‘Cruciferous vegetables’; 1.1 to 5.0% for ‘Legumes’; 1.4 to 5.0% per gram of protein for ‘Other vegetables’ and 2.0 to 5.6% per gram of protein for ‘Starchy roots’. For ‘Fruits and vegetables’, i.e., excluding starchy roots (*n* = 127), the % of protein provided by individual AAs ranged from 1.5 to 4.9% per gram of protein, with the highest contribution from Lys (4.9%), followed by Leu (4.8%), Phe (3.9%), Tyr (2.7%) and Met (1.5%).

### 3.5. Correlation between Protein (Grams) and Amino Acids (mg) per 100 g for Fruits and Vegetables

The correlations between protein (g) and AAs (mg) per 100 g for food samples (*n* = 156) are presented in Table 5. There was a strong correlation found between protein and individual AAs for the ‘Fruits’, ‘Cruciferous vegetables’, ‘Other vegetables’ and ‘Starchy roots’ categories. A moderate association between protein and Met, Leu and Phe was found for ‘Dried fruit’ and ‘Legumes’. In addition, a strong correlation was observed between the individual AAs in different food categories with the exception of Tyr and Met with Lys in ‘Legumes’. The graphs of the correlation between protein content and the individual amino acid content for food categories are presented in Supplement Figure S1.

## 4. Discussion

Fruits and vegetables are crucial for health due to their high content of essential vitamins, minerals and antioxidants [29]. They provide dietary fibre [29], aid digestion [30], are low in calories and fat, and therefore supportive in weight management [31]. The World Health Organisation (WHO) recommends a minimum daily intake of 400 g of fruit and vegetables (excluding potatoes, sweet potatoes, cassava and other starchy roots) as a population-wide goal for those 10 years of age and older [32]. They also suggest intakes of at least 350 g for those aged 6–9 years and 250 g for those aged 2–5 years [32]. A higher intake of fruit and vegetables has been found to reduce the risk of a number of chronic diseases such as cardiovascular disease, type 2 diabetes and cancer [32,33,34]. Starchy roots are an important source of energy in the form of carbohydrates, vitamins, minerals, dietary fibre and phytochemicals [26]. 

Fruits, vegetables and most starchy roots are generally recognised as having a low protein and AA content and are therefore considered beneficial in the dietary management of AA-related IMDs and UCDs [3,14,15]. Data on the AA content of these foods, however, are limited. This can result in the estimation of AA content from protein content rather than using accurate information on the AA content of foods. Some studies have been published that analysed foods in the context of IMDs and these have shown that fruits, vegetables and starchy roots can differ widely in terms of their protein and AA composition [8,9,24,35]. Some of these studies were conducted specifically to determine the Phe content of foods for the management of PKU [8,24], while some analysed Phe alongside other AAs relevant to the management of several IMDs [9,35]. In this study, we present new findings on the protein, Phe, Met, Leu, Lys and Tyr content of a range of fruits, vegetables and starchy roots. The analysis provides new insights into these foods and the various cooking methods selected. This allows inferences to be drawn for use in the dietary management of AA-related IMDs and UCDs. 

Our results showed that the median % protein of ‘Fruits’ was 0.9%. ‘Dried fruits’, on the other hand, had a higher median % protein of 2.2%. In the ‘Vegetable’ groups, the median % protein ranged from 1.3 to 2.8%. The ‘Starchy roots’ had a median % protein of 1.5%. Protein content for individual foods varied considerably. A similar analysis carried out by Ford and colleagues [9] suggested that fruits contain a higher median % of protein (1.9%). However, this included a small sample size of fruits (*n* = 8), half of which were avocados which had a relatively high protein content (1.9 g/100 g). The median % protein of vegetables (2.3%) was similar to our results for ‘Other vegetables’ (2.1%) but higher compared to our results for ‘Cruciferous Vegetables’ (1.3%). Other differences may have arisen in the protein content from the classification of foods into categories. For example, butternut squash, chayote, jackfruit and sundried tomatoes were categorized as ‘Fruits’ in the current study as per the botanical definition of ‘Fruits’ and ‘Vegetables’ [25,27]. The aforementioned publication by Ford and colleagues classed these foods as ‘Vegetables’ as, often in culinary usage, fruits are considered to be sweet while vegetables are more savoury. 

Araujo and colleagues proposed that the Phe content of fruits can be estimated by multiplying their protein content by 3%, i.e., 30 mg of Phe per 1 g of protein. For vegetables, they suggested that a factor of 4%, i.e., 40 mg of Phe per 1 g of protein, may be used [8]. A study by Ford et al. [9] suggested a similar finding of 3% and 4% for fruit and vegetables, respectively. From the present study, the median % of protein provided by Phe for ‘Fruits’ was higher at 4.1% while the vegetable groups had a lower median % Phe of 3.2–3.7%. The ‘starchy roots’ had a median % Phe of 4.6% of protein in comparison to 3% reported by Weetch et al. [24]. Differences may be explained due to variations in the foods analysed, ripeness/maturation of foods at the time of harvest [36,37,38,39,40,41], temperature/climate, soil [42,43], farming methods (organic versus non-organic) [44], storage conditions [45,46] and the system used to classify foods as fruits or vegetables or starchy roots [27]. The median % of the other four AAs investigated in the present study are comparable to those reported in Ford et al. [9] with the exception of % Lys from ‘Fruits’ (3.4% versus 4.8% in our study). It should be highlighted that, within food categories, there was significant variation between % AA per gram of protein for individual foods. Therefore, the AA content of foods should be considered for individual IMDs when deciding if they are allowed freely or need to be counted as part of the natural protein allowance. 

There is some evidence that the method used for cooking foods may impact the amino acid content of foods. For example, Ito and colleagues demonstrated the varying effects of boiling, roasting and microwaving. They reported a decrease in Phe, Met, Leu, Lys and Tyr content of vegetables in most but not all cases when food was boiled [47]. Similarly, a study looking at different cooking methods also found a significant reduction in these five AAs when bamboo shoots were boiled and stir-fried when compared with the steaming method [48]. Foods in our study were cooked prior to the analysis using typical cooking methods to represent the protein and AA content of foods as they are consumed. In some foods where multiple cooking methods were used, e.g., potatoes and sweet potatoes, a lower AA content per 100 g was seen for the majority of food samples when comparing boiling to roasting, baking and air-frying. This lower AA content may be due to the loss of AAs in the cooking water while boiling. A higher overall AA content per 100 g may also be yielded for some cooking methods, e.g., roasting and baking, due to a loss of moisture content from the foods. It was also observed that air-frying tender stem broccoli and baking butternut squash in our study resulted in relatively higher proportions of protein and AAs compared to boiling or steaming, as described by Ford et al. [9]. However, it was noted that the protein content increased while AAs remained similar in parsnips and yams when comparing baking/roasting methods with boiling. Results for green and yellow plantain remained similar for protein and all AAs when boiled and fried. These results indicate that the effects of cooking methods on AA content may vary depending on the type of food, i.e., different foods may have varying structures and compositions that can influence how they respond to cooking methods. No raw samples were analysed for comparison.

Many different food preservation processes can be used to preserve and protect foods from microbial development, for example, canning, freezing, drying, smoking, salting, pickling and vacuum packing. Food preservation methods may impact nutrient content [49,50]. In the current study, the AA content remained largely similar in French beans and spinach when prepared from fresh and frozen foods. This result is interesting and aligns with the general principle of food preservation using freezing and its minimal impact on nutrient stability [51]. This information reinforces the understanding that frozen fruit and vegetables can be a nutritionally viable alternative to fresh options, especially when the same cooking methods are used. 

Some studies have shown that drying food can affect the protein and AA content of the food [52,53]. In our analysis, ‘Dried fruit’ had the lowest % of all five AAs per gram of protein that were investigated. This may be attributed to changes in the AA content in the drying process. Other foods were preserved by canning and pickling in our study, but no fresh versions of these foods were available for comparison. 

In PKU, there is a growing body of evidence which demonstrates that unrestricted consumption of fruit and vegetables with a Phe content of less than 75 mg/100 g does not increase plasma Phe concentrations among patients with PKU [15,16,17,18,19,20,21,22]. The working hypothesis for this finding is that plant proteins have lower digestibility when compared with animal proteins due to an inhibition of the complete absorption of protein, increased endogenous protein losses in the terminal ileum and increased faecal nitrogen excretion caused by the dietary fibre in these foods [54,55]. Therefore, even though fruits and vegetables may provide an increase in Phe in the diet, this does not appear to affect the blood Phe concentrations [18]. As a result of study findings, the European PKU Guidelines recommended that fruits and vegetables (except potatoes) with a Phe content of less than 75 mg/100 g could be safely consumed without measurement by patients with PKU [14]. A recent study by Pinto and colleagues, in 2023, investigated the impact of consuming vegetables with more than 75 mg per 100 g, but did not show convincing results [19]. A limitation of the Pinto study was the fact that children who participated in the extension trial were not adherent, small portions of vegetables were consumed and the range of foods used was limited. Further studies investigating the effects of fruits and vegetables with higher quantities of Phe should be considered amongst the adult PKU population in order for adequate portions to be consumed and investigated. The benefits of simplifying the diet and allowing some fruits and vegetables freely without a negative impact on metabolic control have also been reported for tyrosinaemia [56]. In theory, the simplified diet approach may be advantageous for all AA-related IMDs and UCDs. Internationally, thresholds for protein and amino acid content below which foods can be allowed without restriction need to be considered for these other IMDs.

It is interesting to note that using protein as a proxy of amino acid intake may underestimate foods that can be allowed in the diet of those with AA-related IMDs. For example, foods such as beetroot, butternut squash and mangetout would need to be counted in PKU if using protein values observed but can be allowed freely based on the accurate Phe data obtained in this study.

It is worth noting that the average portion size of fruits, vegetables and starchy roots varies for different foods, and individuals may consume significantly more or less than 100 g of individual foods. For example, rocket contains 85 mg of Phe per 100 g. As per the less than 75 mg Phe per 100 g rule, this requires rocket to be counted as part of the natural protein allowance. However, a typical portion size of rocket as a ‘side salad’ weighs only 10 g and provides 8.5 mg of Phe. This is a good example of a food which contains over 75 mg Phe per 100 g which could potentially be allowed freely in the dietary management of PKU. In contrast, although the average values for avocado fell below the 75 mg Phe per 100 g threshold, given the typical portion size consumed, protein and AA content may need to be considered. 

In the present study, three samples of orange sweet potato were analysed using different cooking methods. The orange sweet potatoes in ‘Batch 2’ had lower protein and AA content compared to batches 1 and 3. This may relate to factors such as ripeness at the time of harvesting, variety and cultivar of the sweet potato, climate, duration and conditions of storage [46]. We were particularly interested in the orange sweet potato results, as its need to be included in the daily natural protein allowance has been debated in our centre in recent years. The practice of counting orange sweet potato as part of the natural protein allowance differs internationally [11,14,23,57]. From our results, although orange sweet potato is lower in protein and individual AAs than standard potatoes, we believe they may need to be considered similarly to standard potatoes which are recommended to be counted as part of the natural protein allowance [14] due to their potential high daily consumption as an energy source. 

This study provides new data on the protein and AA content of a number of foods. We would hope this work could be reproduced in other countries where data on protein and amino acids from fruits, vegetables and starchy roots indigenous to their countries are limited. However, there are a number of limitations that should be highlighted. Due to the high cost of the laboratory analysis, we were unable to reproduce more than one single measurement for protein and individual AA content on each food sample. With the exception of sweet potatoes and avocadoes, additional samples of the same food were not analysed in subsequent test batches. In most cases, foods were prepared using one cooking method only. The foods selected for analysis were dependent on availability and the time of the year. It should also be noted that the Dumas method used in this study to calculate protein content, calculates crude protein content using a conversion factor of 6.25 and assumes that protein contains 16% nitrogen. However, this method does not account for a wide range of compounds that contain non-protein nitrogen [58]. As a result, the Dumas method is not considered wholly accurate for calculating the protein content of foods [59]. It is also important to highlight that portion size norms vary [60,61]. The typical portion sizes in Supplementary Tables S2–S7 are the authors’ perceptions of how much of a given food people would choose to eat. They are not an accurate measurement of average intakes. Therefore, the portion size likely to be consumed warrants consideration and some foods may be allowed within certain limits. 

## 5. Conclusions

Consideration of the protein and AA content of foods is crucial to the dietary management of AA-related IMDs and UCDs. This study presents data on the protein and AA analysis of 165 fruits, vegetables and starchy roots sampled. These results add to existing research publications that focus on protein and AA content of foods within the context of IMDs. This study included many foods and methods of cooking for which data were either limited or non-existent. Our results demonstrate that cooking methods can significantly affect the protein and AA content of some but not all foods, with air-frying, roasting and baking yielding a higher AA content when compared with boiling. This study could potentially lead to more fruits and vegetables being identified and recommended as foods that can be allowed freely without measurement in the diets of those with AA-related IMDs and UCDs. This would, thereby, increase the variety and nutrient intake of a patient’s diet and allow for the achievement of the WHO minimum targets for fruit and vegetables recommended for the general population. It may also help to ease some of the burdens of following a restrictive, low natural protein diet. It can be used to identify foods with a higher protein and AA content which need to be restricted and counted as part of the natural protein allowance. Information from this study may assist dietitians working with those with AA-related IMDs and UCDs and result in improved metabolic control for patients.

## Figures and Tables

**Table 1:** Protein and amino acid content per 100 g of edible portion of each food.

			Amino Acids mg/100 g				
Food	Category	Protein g/100 g	Phe	Met	Leu	Lys	Tyr
Apricot, dried	Dried fruit	2.9	54	19	69	77	42
Apricot, raw	Fruit	0.5	23	5	22	19	11
Artichoke, globe, boiled	Other vegetables	2.2	85	39	137	137	62
Artichoke, grilled, jarred	Other vegetables	2.0	75	29	109	103	58
Artichoke, jerusalem, boiled	Other vegetables	1.3	60	21	68	93	36
Asparagus, boiled	Other vegetables	2.7	80	33	110	108	59
Aubergine (eggplant), grilled	Fruit	1.2	38	11	37	47	25
Aubergine, Thai (thai eggplant), sautéed	Fruit	1.3	57	20	68	70	37
Aubergine, white (white eggplant), grilled	Fruit	2.3	84	28	103	108	60
Avocado, Haas, raw	Fruit	1.3	59	23	83	76	36
Avocado, raw	Fruit	1.1	56	21	74	72	33
Baby corn, boiled	Other vegetables	1.8	76	29	102	108	56
Bamboo shoots, tinned	Other vegetables	0.7	41	15	57	55	33
Banana, dried ^a^	Dried fruit	2.0	78	27	85	80	39
Banana, raw	Fruit	1.3	43	18	66	58	22
Beetroot, boiled	Other vegetables	2.5	46	20	66	90	45
Bitter gourd (bitter melon/karela), boiled	Fruit	1.2	52	17	62	61	39
Black kale (cavolo nero), boiled	Cruciferous vegetables	3.3	133	44	229	193	108
Black radish, raw	Cruciferous vegetables	1.3	38	18	52	69	25
Blackberries, raw	Fruit	1.7	46	13	60	59	31
Blueberries, raw	Fruit	0.3	22	7	22	19	12
Broad beans, frozen, boiled	Legumes	6.8	287	60	467	481	186
Broccoli, steamed	Cruciferous vegetables	3.5	99	46	148	220	83
Broccoli, tenderstem (broccolini), airfried	Cruciferous vegetables	6.5	201	73	252	270	167
Broccoli microgreens, raw	Cruciferous vegetables	2.5	85	30	107	109	58
Brussel sprouts, frozen, boiled	Cruciferous vegetables	2.3	79	32	109	154	59
Burdock root (gobo), roasted	Starchy roots	5.4	141	28	106	134	63
Butternut squash, baked	Fruit	3.9	62	44	61	71	64
Cabbage, napa (Chinese leaf), boiled	Cruciferous vegetables	1.0	37	15	44	42	23
Cabbage, red, boiled	Cruciferous vegetables	1.1	37	14	46	62	28
Cabbage, savoy, boiled	Cruciferous vegetables	0.7	29	11	33	38	21
Cabbage, sweetheart, boiled	Cruciferous vegetables	1.0	38	19	48	54	24
Cabbage, white, boiled	Cruciferous vegetables	0.9	33	11	38	54	25
Cabbage, york, boiled	Cruciferous vegetables	0.8	41	17	54	75	35
Cauliflower rice, frozen, microwaved	Cruciferous vegetables	1.2	55	24	78	107	38
Cauliflower, steamed	Cruciferous vegetables	1.7	70	32	103	143	49
Celeriac, boiled	Other vegetables	0.7	27	11	29	38	19
Chayote (chow chow), boiled	Fruit	0.5	23	7	21	22	15
Chayote (chow chow), raw	Fruit	0.8	27	9	30	34	23
Chicory, red, raw	Other vegetables	1.4	33	12	37	33	21
Chicory, yellow, raw	Other vegetables	1.0	27	11	29	29	16
Choy sum, steamed	Cruciferous vegetables	2.3	82	34	121	114	42
Coconut, desiccated	Dried fruit	7.1	278	117	378	324	175
Cornichons, jarred, pickled	Fruit	1.5	103	22	130	243	133
Courgette (zucchini), green, lightly fried	Fruit	0.9	38	17	41	46	22
Cranberries, dried ^b^	Dried fruit	1.0	22	9	21	14	9
Curly kale, boiled	Cruciferous vegetables	2.4	100	39	142	149	79
Currants, dried	Dried fruit	2.0	62	20	78	69	26
Custard apple, raw	Fruit	2.3	54	19	69	69	36
Dates, dried	Dried fruit	1.9	55	16	69	55	32
Dates, Medjool, dried	Dried fruit	2.2	72	20	93	60	41
Dok kae flower (Karturat flower), steamed	Other vegetables	2.3	105	46	169	165	81
Dragon fruit, red, raw	Fruit	0.8	61	32	73	60	46
Dragon fruit, yellow, raw	Fruit	1.0	48	27	53	41	32
Drumsticks, boiled	Other vegetables	1.9	54	24	81	71	34
Fennel, raw	Other vegetables	0.9	30	10	32	31	20
Figs, dried	Dried fruit	3.2	123	19	159	109	72
Figs, raw	Fruit	1.0	41	14	51	49	26
French beans, fresh, boiled	Legumes	2.2	76	28	106	119	62
French beans, frozen, boiled	Legumes	1.8	78	27	109	122	57
Gherkins, jarred, pickled	Fruit	0.9	49	15	56	51	30
Goji berries, dried	Dried fruit	11.5	187	67	316	203	128
Jackfruit, tinned	Fruit	1.8	48	15	65	65	33
Jackfruit, young green, tinned	Fruit	0.5	34	11	41	40	22
Jalapeno peppers, pickled, jarred	Fruit	0.5	27	9	25	21	11
Kai lan (Chinese broccoli), boiled	Cruciferous vegetables	2.6	98	29	106	104	51
Kiwi, raw	Fruit	1.0	41	21	52	54	30
Kohlrabi, boiled	Cruciferous vegetables	0.5	18	7	17	20	11
Korean pear, raw	Fruit	0.4	13	6	13	8	<5
Kumquats, raw	Fruit	1.6	50	14	63	68	38
Leeks, sauteed	Other vegetables	1.0	43	15	56	75	27
Longans, tinned	Fruit	0.7	26	11	33	35	18
Lotus root, boiled	Starchy roots	1.6	62	20	62	64	38
Lychees, tinned	Fruit	0.7	37	14	44	43	19
Mandarins, raw	Fruit	0.6	23	7	20	24	15
Mangetout, steamed	Legumes	3.6	71	25	96	130	48
Mango, dried	Dried fruit	1.9	85	46	111	99	45
Mango, raw	Fruit	0.3	17	9	19	23	10
Mangosteen, raw	Fruit	0.6	30	11	38	36	16
Mooli (daikon), green, raw	Cruciferous vegetables	1.0	24	9	24	39	15
Mooli (daikon), white, raw	Cruciferous vegetables	2.6	43	17	56	73	23
Mushrooms, beech (shimoji brown), lightly fried	Other vegetables	2.3	77	26	115	104	57
Mushrooms, beech (shimoji white), lightly fried	Other vegetables	4.1	119	41	186	207	86
Mushrooms, button, lightly fried	Other vegetables	3.5	88	30	117	139	67
Mushrooms, chestnut, lightly fried	Other vegetables	2.8	79	29	112	109	59
Mushrooms, closed cap, lightly fried	Other vegetables	3.5	91	39	149	149	79
Mushrooms, oyster, lightly fried	Other vegetables	2.7	99	34	145	152	60
Mushrooms, portobello, lightly fried	Other vegetables	2.8	78	34	112	108	70
Mushrooms, shitake, lightly fried	Other vegetables	3.5	101	34	150	150	76
Mustard greens, boiled	Cruciferous vegetables	0.7	30	16	38	41	19
Nectarines, raw	Fruit	0.9	23	6	26	36	18
Orange, raw	Fruit	0.8	21	10	24	31	14
Pak choi (bok choy), steamed	Cruciferous vegetables	1.4	44	13	53	70	7
Palm seeds, tinned	Fruit	<0.1	10	<5	<5	<5	<5
Papaya, raw	Fruit	0.5	23	7	21	19	16
Parsnip, boiled	Other vegetables	1.0	57	20	71	81	38
Parsnip, roasted	Other vegetables	1.4	54	20	67	75	35
Passion fruit, raw	Fruit	2.0	123	32	109	99	49
Pea aubergine, sauteed	Fruit	2.5	96	34	129	120	53
Peaches, raw	Fruit	1.1	22	<5	24	27	23
Peppers (capsicum), green, raw	Fruit	0.6	21	7	22	23	16
Peppers (capsicum), red, raw	Fruit	0.7	25	11	25	26	17
Peppers (capsicum), yellow, raw	Fruit	0.6	23	9	22	26	19
Persimmon (sharon fruit), raw	Fruit	0.8	30	11	32	34	15
Physallis (cape gooseberry/groundcherry), raw	Fruit	1.4	61	27	81	68	42
Plaintain, green, boiled	Starchy roots	1.1	59	22	72	70	35
Plaintain, green, fried	Starchy roots	1.5	60	22	73	76	33
Plaintain, yellow, boiled	Starchy roots	1.2	61	20	68	68	32
Plaintain, yellow, fried	Starchy roots	1.1	55	17	69	55	29
Pomegranate, seeds only, raw	Fruit	1.4	83	35	118	70	63
Potatoes, baby, baked	Starchy roots	2.2	97	38	131	124	79
Potatoes, baby, boiled	Starchy roots	1.8	82	31	105	112	70
Potatoes, baby, roasted	Starchy roots	2.3	101	37	133	135	86
Potatoes, Maris Piper, airfried	Starchy roots	2.6	111	58	133	148	90
Potatoes, Maris Piper, baked	Starchy roots	1.9	72	38	91	96	59
Potatoes, Maris Piper, boiled	Starchy roots	1.2	51	26	63	67	38
Potatoes, Maris Piper, mashed with butter	Starchy roots	1.1	51	24	68	53	41
Potatoes, Maris Piper, roasted	Starchy roots	1.9	82	38	115	109	61
Potatoes, Rooster, airfried	Starchy roots	2.9	112	65	150	168	102
Potatoes, Rooster, baked	Starchy roots	2.6	116	57	140	153	109
Potatoes, Rooster, boiled	Starchy roots	1.3	61	31	77	75	55
Potatoes, Rooster, roasted	Starchy rootss	1.7	80	37	117	115	67
Potatoes, white, baked	Starchy rootss	1.9	82	34	90	93	59
Potatoes, white, boiled	Starchy roots	1.4	64	29	67	67	43
Potatoes, white, mashed with butter	Starchy rootss	1.3	63	28	62	60	46
Potatoes, white, roasted	Starchy roots	2.0	82	40	82	92	68
Prunes, dried	Dried fruit	1.9	54	15	60	35	26
Pumpkin (skin on), roasted	Fruit	2.2	81	30	118	128	68
Quince, raw	Fruit	0.3	16	8	20	21	9
Raddicchio, raw	Other vegetables	0.8	37	16	48	32	24
Raisins, dried	Dried fruit	2.4	59	16	77	70	31
Rambutan, tinned	Fruit	0.5	30	10	33	40	21
Redcurrants, raw	Fruit	1.7	62	25	77	83	29
Rhubarb, champagne, boiled	Other vegetables	1.1	35	15	44	51	28
Rocket (argula), raw	Cruciferous vegetables	3.0	85	31	104	92	52
Runner beans, boiled	Legumes	1.4	41	17	58	61	29
Snakefruit, raw	Fruit	0.5	26	13	35	32	15
Spinach, fresh, boiled	Other vegetables	3.0	123	49	183	168	110
Spinach, frozen, boiled	Other vegetables	2.6	137	55	201	180	106
Starfruit (carambola), raw	Fruit	0.8	25	11	32	40	26
Sugar snap peas, boiled	Legumes	3.4	76	26	107	136	55
Sultanas, dried	Dried fruit	2.8	67	17	85	76	33
Swede (rutabaga), boiled	Cruciferous vegetables	0.6	23	8	29	44	16
Sweet potato, orange, airfried, batch 3	Starchy roots	1.3	85	27	79	69	40
Sweet potato, orange, baked, batch 1	Starchy roots	1.3	55	23	65	51	35
Sweet potato, orange, baked, batch 2	Starchy roots	0.8	53	22	53	51	28
Sweet potato, orange, baked, batch 3	Starchy roots	1.5	91	30	88	76	49
Sweet potato, orange, boiled, batch 1	Starchy roots	1.0	46	16	54	46	29
Sweet potato, orange, boiled, batch 2	Starchy roots	0.6	39	16	42	45	22
Sweet potato, orange, boiled, batch 3	Starchy roots	1.1	69	25	68	59	41
Sweet potato, orange, roasted, batch 1	Starchy roots	1.8	110	40	106	89	74
Sweet potato, orange, roasted, batch 2	Starchy roots	1.0	45	17	45	41	23
Sweet potato, orange, roasted, batch 3	Starchy roots	1.7	104	37	104	90	59
Sweet potato, purple, baked	Starchy roots	2.4	128	39	123	92	74
Sweet potato, purple, boiled	Starchy roots	1.4	67	28	92	87	45
Sweet potato, white, boiled, batch 1	Starchy roots	1.5	82	27	89	80	47
Sweet potato, white, boiled, batch 2	Starchy roots	0.6	37	14	38	37	24
Sweet potato, white, baked, batch 2	Starchy roots	0.6	39	9	28	25	16
Tamarind, boiled	Fruit	1.1	61	19	64	53	28
Taro, boiled	Starchy roots	0.9	49	13	62	59	38
Tomato, sundried, jarred	Fruit	3.4	124	27	102	124	65
Vine leaves, boiled	Other vegetables	3.5	170	76	253	241	128
Water chestnuts, tinned	Other vegetables	0.6	30	16	45	37	26
Yam, baked	Starchy roots	1.5	29	11	30	35	26
Yam, boiled	Starchy roots	0.8	26	9	27	32	16

^a^ Banana, dried: Contain banana, coconut oil, cane sugar, honey; ^b^ Cranberries: apple juice infused, contain 60% dried cranberries (60%), apple juice concentrate (39%) sunflower oil (1%).

**Table 2:** The amount of protein (g) and amino acids (mg) per 100 g of edible portion of food categories.

Food Category	Protein	Phe	Met	Leu	Lys	Tyr
	(g/100 g)	(mg/100 g)	(mg/100 g)	(mg/100 g)	(mg/100 g)	(mg/100 g)
Fruit (*n* = 50) a,b						
Mean ± SD	1.1 ± 0.9	45 ± 27	16 ± 9	53 ± 32	54 ± 39	30 ± 22
Median [range]	0.9 [0.3–3.9]	37 [13–124]	14 [5–44]	43 [13–130]	45 [8–243]	24 [9–133]
Dried fruit (*n* = 13)						
Mean ± SD	3.3 ± 2.9	92 ± 69	32 ± 30	123 ± 105	98 ± 82	54 ± 47
Median [range]	2.2 [1.0–11.5]	67 [21–278]	19 [9–117]	85 [21–378]	76 [14–324]	39 [9–175]
Cruciferous vegetables (*n* = 24)						
Mean ± SD	1.9 ± 1.3	63 ± 43	24 ± 15	85 ± 62	97 ± 63	44 ± 36
Median [range]	1.3 [0.5–6.5]	44 [18–201]	19 [7–73]	55 [17–251]	74 [20–270]	31 [7–167]
Legumes (*n* = 6)						
Mean ± SD	3.1 ± 2.0	105 ± 90	31 ± 15	157 ± 153	175 ± 153	73 ± 56
Median [range]	2.8 [1.4–6.8]	76 [41–287]	36 [17–60]	107 [58–467]	126 [61–481]	56 [29–186]
Other vegetables (*n* = 30)						
Mean ± SD	2.1 ± 1.0	72 ± 36	28 ± 15	103 ± 58	104 ± 56	54 ± 29
Median [range]	2.1 [0.6–4.1]	75 [27–170]	27 [10–76]	105 [29–253]	98 [29–241]	56 [16–128]
Starchy roots (*n* = 33) c						
Mean ± SD	1.6 ± 0.8	74 ± 27	29 ± 13	84 ± 32	84 ± 36	52 ± 23
Median [range]	1.5 [0.6–5.4]	66 [26–141]	28 [9–65]	77 [27–150]	73 [25–168]	45 [16–109]

Abbreviations: SD, standard deviation; ^a^ Reported protein data < 0.1 g/100 g and/or amino acid data < 5 mg/ 100 g were excluded from the analysis; ^b^ Average values were taken for two samples of avocado; ^c^ Average values were taken for three samples of sweet potato (orange, baked), sweet potato (orange, boiled), sweet potato (orange, roasted) and two samples of sweet potato (white, boiled).

**Table 3:** Milligrams of amino acids per gram of protein of food categories.

Food Category	mg Phe	mg Met	mg Leu	mg Lys	mg Tyr
Fruit (*n* = 50) ^a, b^					
Mean ± SD	43 ± 14	16 ± 7	50 ± 17	51 ± 22	28 ± 13
Median [range]	41 [16–80]	15 [7–42]	50 [16–97]	48 [18–168]	27 [15–92]
Dried fruit (*n* = 13)					
Mean ± SD	30 ± 9	10 ± 5	38 ± 12	30 ± 11	16 ± 5
Median [range]	29 [16–45]	8 [6–25]	36 [20–59]	29 [13–53]	15 [9–25]
Cruciferous vegetables (*n* = 24)					
Mean ± SD	36 ± 7	14 ± 4	46 ± 12	56 ± 17	24 ± 8
Median [range]	37 [16–48]	14 [7–21]	45 [22–70]	55 [29–93]	25 [5–42]
Legumes (*n* = 6)					
Mean ± SD	32 ± 10	11 ± 3	47 ± 16	53 ± 15	23 ± 7
Median [range]	32 [20–44]	11 [7–15]	45 [27–69]	50 [37–71]	24 [14–32]
Other vegetables (*n* = 30)					
Mean ± SD	38 ± 11	15 ± 4	51 ± 15	51 ± 15	27 ± 8
Median [range]	37 [18–63]	14 [8–26]	47 [26–79]	50 [24–78]	26 [16–46]
Starchy roots (*n* = 33) ^c^					
Mean ± SD	47 ± 9	18 ± 4	53 ± 12	52 ± 10	32 ± 7
Median [range]	46 [20–65]	20 [5–25]	56 [20–70]	53 [24–69]	33 [12–44]

^a^ Reported protein data < 0.1 g/100 g and/or amino acid data < 5 mg/100 g were excluded from the analysis; ^b^ Average values were taken for two samples of avocado; ^c^ Average values were taken for three samples of sweet potato (orange, baked), sweet potato (orange, boiled), sweet potato (orange, roasted) and two samples of sweet potato (white, boiled).

**Table 4:** Percent of amino acids per gram of protein of food categories.

Food Category	mg Phe	mg Met	mg Leu	mg Lys	mg Tyr
Fruit (*n* = 50) ^a,b^					
Mean ± SD	4.3 ± 1.4	1.6 ± 0.7	5.0 ± 1.7	5.1 ± 2.2	2.8 ± 1.3
Median [range]	4.1 [1.6–8.0]	1.5 [0.7–4.2]	5.0 [1.6–9.7]	4.8 [1.8–16.8]	2.7 [1.5–9.2]
Dried fruit (*n* = 13)					
Mean ± SD	3.0 ± 0.9	1.0 ± 0.5	3.8 ± 1.2	3.0 ± 1.1	1.6 ± 0.5
Median [range]	2.9 [1.6–4.5]	0.8 [0.6–2.5]	3.6 [2.0–5.9]	2.9 [1.3–5.3]	1.5 [0.9–2.5]
Cruciferous vegetables (*n* = 24)					
Mean ± SD	3.6 ± 0.7	1.4 ± 0.4	4.6 ± 1.2	5.6 ± 1.7	2.4 ± 0.8
Median [range]	3.7 [1.6–4.8]	1.4 [0.7–2.1]	4.5 [2.2–7.0]	5.5 [2.9–9.3]	2.5 [0.5–4.2]
Legumes (*n* = 6)					
Mean ± SD	3.2 ± 1.0	1.1 ± 0.3	4.7 ± 1.6	5.3 ± 1.5	2.3 ± 0.7
Median [range]	3.2 [2.0–4.4]	1.1 [0.7–1.5]	4.5 [2.7–6.9]	5.0 [3.7–7.1]	2.4 [1.4–3.2]
Other vegetables (*n* = 30)					
Mean ± SD	3.8 ± 1.1	1.5 ± 0.4	5.1 ± 1.5	5.1 ± 1.5	2.7 ± 0.8
Median [range]	3.7 [1.8–6.3]	1.4 [0.8–2.6]	4.7 [2.6–7.9]	5.0 [2.4–7.8]	2.6 [1.6–4.6]
Starchy roots (*n* = 33) ^c^					
Mean ± SD	4.6 ± 0.9	1.8 ± 0.4	5.3 ± 1.5	5.1 ± 1.5	3.2 ± 0.7
Median [range]	4.6 [2.0–6.5]	2.0 [0.5–2.5]	5.6 [2.0–7.0]	53 [2.4–6.9]	3.3 [1.2–4.4]

^a^ Reported protein data < 0.1 g/100 g and/or amino acid data < 5 mg/100 g were excluded from this analysis; ^b^ Average values were taken for two samples of avocado; ^c^ Average values were taken for three samples of sweet potato (orange, baked), sweet potato (orange, boiled), sweet potato (orange, roasted) and two samples of sweet potato (white, boiled).

**Table 5:** Correlation between protein (g) and amino acid (mg) per 100 g for fruits, vegetables and starchy roots.

Food Category	Protein	Phe	Met	Leu	Lys	Tyr
Fruits (*n* = 50) ^a,b^						
Protein	-					
Phe	0.85	-				
Met	0.78	0.93	-			
Leu	0.85	0.97	0.92	-		
Lys	0.9	0.95	0.9	0.97	-	
Tyr	0.88	0.91	0.86	0.91	0.93	-
Dried fruit (*n* = 13)						
Protein	-					
Phe	0.6	-				
Met	0.53	0.82	-			
Leu	0.65	0.99	0.85	-		
Lys	0.75	0.85	0.8	0.85	-	
Tyr	0.75	0.82	0.81	0.86	0.91	-
Cruciferous vegetables (*n* = 24)						
Protein	-					
Phe	0.91	-				
Met	0.84	0.93	-			
Leu	0.89	0.98	0.96	-		
Lys	0.85	0.94	0.94	0.97	-	
Tyr	0.79	0.89	0.92	0.91	0.91	-
Legumes (*n* = 6)						
Protein	-					
Phe	0.46	-				
Met	0.47	0.9	-			
Leu	0.49	0.99	0.83	-		
Lys	0.89	0.64	0.49	0.71	-	
Tyr	0.49	0.9	1	0.83	0.47	-
Other vegetables (*n* = 30)						
Protein	-					
Phe	0.87	-				
Met	0.84	0.97	-			
Leu	0.86	0.99	0.97	-		
Lys	0.87	0.98	0.95	0.97	-	
Tyr	0.89	0.98	0.98	0.98	0.97	-
Starchy roots (*n* = 33) ^c^						
Protein	-					
Phe	0.89	-				
Met	0.85	0.86	-			
Leu	0.84	0.89	0.88	-		
Lys	0.88	0.87	0.88	0.94	-	
Tyr	0.87	0.88	0.95	0.87	0.9	-

^a^ Reported protein data of < 0.1 g/100 g and/or amino acid data < 5 mg/100 g were excluded from the analysis; ^b^ Average values were taken for two samples of avocado; ^c^ Average values were taken for three samples of sweet potato (orange, baked), sweet potato (orange, boiled), sweet potato (orange, roasted) and two samples of sweet potato (white, boiled).

## Data Availability

The data presented in this study are available on request from the corresponding author.

## Supplementary Materials

The following supporting information can be downloaded at: https://www.mdpi.com/article/10.3390/nu16172812/s1, Table S1: Methods used for preparing fruit and vegetables; Table S2: The protein content of food samples based on ‘typical’ portion sizes in grams (g); Table S3: The Phenylalanine (Phe) content of food samples based on ‘typical’ portion sizes in milligrams (mg); Table S4: The methionine (Met) content of food samples based on ‘typical’ portion sizes in milligrams (mg); Table S5: The leucine (Leu) content of food samples based on ‘typical’ portion sizes in milligrams (mg); Table S6: The lysine (Lys) content of food samples based on ‘typical’ portion sizes in milligrams (mg); Table S7: The tyrosine (Tyr) and phenylalanine (Phe) content of food samples based on ‘typical’ portion sizes in milligrams (mg). Figure S1: Correlation between protein (g) and amino acid (mg) per 100 g for fruits, vegetables and starchy roots.
