# Supplementary material for: Determination of the Protein and Amino Acid Content of Fruit, Vegetables and Starchy Roots for Use in Inherited Metabolic Disorders
Source: Nutrients. 2024 Aug 23;16(17):2812. doi: 10.3390/nu16172812 (PMC11397706; doi:10.3390/nu16172812)
Supplement: Supplementary file 1 [file nutrients-16-02812-s001.zip › Table S1 Preparation of food samples.pdf]

Table S1: Methods used for preparing fruit and vegetables

Food	Instructions
Apricot, dried	Stoneless. Dried, weigh out quantity required.
Apricot, raw	Fresh, raw, skin on. Halve apricot to remove stone and slice, weigh out raw quantity required.
Artichoke, globe, boiled	Cut stalk off. Remove the tough leaves close to the base of the artichokes. Cut off the stems completely, so the artichokes will sit flat. Cook in boiling water. It will take 15-30 minutes, depending on the size and freshness of the artichoke, so check from 15 minutes onwards. The best test of doneness is when a leaf from the middle pulls away easily. Drain well.
Artichoke, grilled, jarred	Drain well, weigh out quantity required.
Artichoke, jerusalem, boiled	Soak the artichokes in cold water for 20 minutess or so to loosen any dirt, then scrub them with a scourer, being sure to remove any grit. Halve the small ones and quarter the bigger ones, add cold water and bring to the boil until soft like potato. This can take 10-30 minutes. Drain well.
Asparagus, boiled	Trim woody parts of stalk and boil for 3-5 minutes.
Aubergine (eggplant), grilled	Cut the aubergines into slices or cubes. Brush with oil and grill under moderate heat until golden.
Aubergine, Thai (thai eggplant), sauteed	Cut the Thai aubergines into slices, cubes, strips, or halves. Sweat in frying pan to reduce moisture for a few minutes.
Aubergine, white (white eggplant), grilled	Cut the aubergine into slices or cubes. Brush with oil and grill under moderate heat until golden.
Avocado, Haas, raw	Halve, remove stone and scoop flesh and measure out required quantity.
Avocado, raw	Halve, remove stone and scoop flesh and measure out required quantity.
Baby corn, boiled	Boil in unsalted water until tender.
Bamboo shoots, tinned	Drain and chop enough for required quantity.
Banana, dried	Ingredients: banana, coconut oil, cane sugar, honey. Weigh out required quantity.
Banana, raw	Remove skin.
Beetroot, boiled	Wash the beets gently, making sure not to tear the skins, then place in a pan of warm water, bring to the boil, reduce the heat and simmer until tender – around 20-30 minutes.
Bitter gourd (bitter melon/karela), boiled	Halve, remove the seeds and then slice it crosswise or boil the halves. Place the bitter melon in a large pot and just cover with water. Cook over medium heat until tender for about 10 to 15 minutes.
Black kale (cavolo nero), boiled	Bring a large pan of salted water to the boil. Drop in the cavalo nero and cook for 3-4 minutes until wilted and darker in colour. Drain well.
Black radish, raw	Wash, leave skin on and slice/dice.
Blackberries, raw	Raw as they are.
Blueberries, raw	Raw as they are.
Broad beans, frozen, boiled	Place in a saucepan of boiling water. Bring back to the boil. Simmer for 5-6 minutes. Drain well before serving.
Broccoli, steamed	Separate broccoli into florets and steamed for 6-8 minutes
Broccoli, tenderstem (broccolini), airfried	Wash the broccoli. Pat dry. Place in the airfrier and set to 180 C. Once your airfrier is heated, cook for 5 minutes.
Broccoli microgreens, raw	Raw as they are.
Brussel sprouts, frozen, boiled	Boil in unsalted water until tender as per packet.
Burdock root (gobo), roasted	Preheat the oven to 200°C. Burdock root is scrubbed and peeled with a peeler or back of a knife (careful not to peel to much) before being cut into rounds and tossed up with 1 tablespoon of olive oil. Once spread out on a baking sheet, oven roast for about 15 minutes, or until golden on one side, then flip. Return to the oven for another 10 minutes or so.

Appendix 1. *Continued*

**Food**	**Instructions**
Butternut squash, baked	Preheat oven to 200°C. Use a sharp knife to cut off the top of the butternut squash then hold the base of the squash and carefully cut in half. Using a peeler, remove all the skin from the squash. Cut the squash in half and use a plastic spoon and remove the seeds. Using a knife cut the squash into even slices. Arrange the seasoned cubes on a baking sheet and bake in the preheated oven until the squash is lightly browned and tender (about 25-30 minutes.
Cabbage, napa (Chinese leaf), boiled	Remove any outer layers of the cabbage that seem tough or are not fresh and discard them. With a large chef's knife, slice off the root of the cabbage, cut the halves into quarters, shred by cutting diagonally across the cabbage wedges into thin slices. Drop into a pot of boiling water. Boil shredded cabbage for 12 to 15 minutes.
Cabbage, red, boiled	Remove any outer layers of the cabbage that seem tough or are not fresh and discard them. With a large chef's knife, slice off the root of the cabbage, cut the halves into quarters, shred by cutting diagonally across the cabbage wedges into thin slices. Drop into a large pot of boiling water and shredded cabbage takes 12 to 15 minutes to boil.
Cabbage, savoy, boiled	Remove any outer layers of the cabbage that seem tough or are not fresh and discard them. With a large chef's knife, slice off the root of the cabbage, cut the halves into quarters, shred by cutting diagonally across the cabbage wedges into thin slices. Drop into a large pot of boiling water and shredded cabbage takes 12 to 15 minutes to boil.
Cabbage, sweetheart, boiled	Remove any outer layers of the cabbage that seem tough or are not fresh and discard them. With a large chef's knife, slice off the root of the cabbage, cut the halves into quarters, shred by cutting diagonally across the cabbage wedges into thin slices. Drop into a large pot of boiling water and shredded cabbage takes 12 to 15 minutes to boil.
Cabbage, white, boiled	Remove any outer layers of the cabbage that seem tough or are not fresh and discard them. With a large chef's knife, slice off the root of the cabbage, cut the halves into quarters, shred by cutting diagonally across the cabbage wedges into thin slices. Drop into a large pot of boiling water and shredded cabbage takes 12 to 15 minutes to boil.
Cabbage, york, boiled	Remove any outer layers of the cabbage that seem tough or are not fresh and discard them. With a large chef's knife, slice off the root of the cabbage, cut the halves into quarters, shred by cutting diagonally across the cabbage wedges into thin slices. Drop into a large pot of boiling water and shredded cabbage takes 12 to 15 minutes to boil.
Cauliflower rice, frozen, microwaved	Place cauliflower rice bag, unopened, printed side up in microwave. (Do not pierce steambag). Adjust cooking time according to the power rating of your microwave oven and the serving quantity. 1 bag: 650W 3 ½ minutes; 750W 3 ½ minutes; 850W 3 minutes. Stand for 1 minute. Remove bag from microwave, drain.
Cauliflower, steamed	Remove leaves. Separate cauliflower into florets and steam for 5-10 minutes.
Celeriac, boiled	Shave off ends and use knife to peel outside skin, cutting off cracks, chop up into slices or cubes, boiled in unsalted water for 15-20 minutes.
Chayote (chow chow), boiled	Peel like a potato. Cut and deseed by slicing in half lengthways, pop seeds out with spoon, chop up into chunks, boil until tender.
Chayote (chow chow), raw	Peel like a potato. Cut and deseed by slicing in half lengthways, pop seeds out with spoon, chop up the flesh into chunks.
Chicory, yellow, raw	Wash and clean leaves, cut the stalks from the leaves and leave whole or chop as required.

Appendix 1. *Continued*

**Food**	**Instructions**
Chicory, red, raw	Wash and clean leaves, cut the stalks from the leaves and leave whole or chop as required.
Choy sum, steamed	Rinse leaves and stem, cut off any woody parts of stem, and steam until tender for about 5-8 minutes.
Coconut, desiccated	Dried, weigh out quantity required.
Cornichons, jarred, pickled	Drain and weigh out quantity required.
Courgette (zucchini), green, lightly fried	Chop off each end, cut in half lengthwise and slice. Lightly fry using a small amount of oil until golden.
Cranberries, dried	Apple juice infused, 60 % dried Cranberries (60%), apple juice concentrate (39%) sunflower Oil (1%)). Dried, weigh out quantity required.
Curly kale, boiled	Bring the water to a boil in a large wide saucepan. Add the kale leaves and cook uncovered at a good simmer for about 8 minutes. Taste a small piece of the kale to ensure it is tender. Drain the kale and allow to sit in a sieve or colander to lose as much of the cooking water as possible.
Currants, dried	Dried, weigh out quantity required.
Custard apple, raw	Cut in half, discard the seeds and scoop out the flesh.
Dates, dried	Stones removed. Dried, weigh out quantity required.
Dates, Medjool, dried	Stones removed. Dried, remove stone and weigh out quantity required.
Dok kae flower (Karturat flower), raw	Steam until tender for 5-6 minutes.
Dragon fruit, red, raw	Use a large knife to cut in half lengthways. The flesh of the dragon fruit is red and has tiny black edible seeds. Use a spoon to scoop out the flesh. Use a sharp knife to trim any skin that is still attached.
Dragon fruit, yellow, raw	Use a large knife to cut in half lengthways, the flesh of the dragon fruit is white and has tiny black edible seeds. Use a spoon to scoop out the flesh. Use a sharp knife to trim any skin that is still attached.
Drumsticks, boiled	Wash. Cut the drumstick into 5 cm pieces. Boil a saucepan of water. Add the drumstick pieces and cook on a medium heat for 8-10 minutes or until they turn soft. Drain.
Fennel, raw	Wash, then trim off the green tops, slice off the shoots and root and peel off the tougher outer layer (if the bulb is particularly young and tender you can leave this layer on). Chop into quarters and remove the core from each one (but not too much, or the quarters will fall apart).
Figs, dried	Dried, weigh out quantity required.
Figs, raw	Raw flesh and skin, gently wipe the skins with a damp cloth, trim off the stem if it is hard, cut in half from top to bottom, slice into wedges.
French beans, fresh, boiled	Rinse and trim the beans, bring to the boil and simmer for 5-8 minutes (may need longer if thick).
French beans, frozen, boiled	Boil in unsalted water until tender as per packet.
Gherkins, jarred, pickled	Drain and weigh.
Goji berries, dried	Dried, weigh out quantity required.
Jackfruit, tinned	Drain and weigh.
Jackfruit, young green, tinned	Drain and weigh.
Jalapeno peppers, pickled, jarred	Drain and rinse, dry and weigh out quantity required.

Appendix 1. *Continued*

Food	Instructions
Kai lan (Chinese broccoli), boiled	Remove any grit from leaves. Trim about a half inch from the bottom of the stalks. Rinse with cold running water, then pat dry. If the Kai Lan is small, you can leave it whole. If larger, cut the stalks into 2- to 4-cm lengths, slice the leaves down the centre, then cut across into ribbons. Bring water to a boil in a large saucepan. Immerse for 30 seconds for small stalks and no more than a minute for larger stalks. Drain and refresh under cold running water to retain colour.
Kiwi, raw	Cut off both ends of the kiwi with a sharp serrated knife. Slide the bowl of a large spoon as close to the skin as you can and rotate the spoon to scoop the flesh out.
Kohlrabi, boiled	Snip off the leaf stems. Trim off the base and top and then use a potato peeler or sharp knife to peel it as if it's an apple. Then thinly slice, chunk or cut into wedges. Boil for up to 12 minutes.
Korean pear, raw	Cut the pear in half. Slice the pear halves into halves. Cut down diagonally to remove the core (the seeds are not for eating) and peel the outer skin off the slices. Weigh required amount.
Kumquats, raw	Wash, pull off the stem, then chop or slice as required. The thin skin is generally left on. Weigh out required quantity.
Leeks, sauteed	Slice leeks in half lengthwise. Chop off stringy roots and dark green leaves, continue chopping the white and light green parts, cutting into half-moons, leeks can be dirty inside, so add to colander and rinse under cool running water, use paper towel to dry before cooking. Saute in a little oil for 5 minutes, stirring occasionally until softened.
Longans, tinned	Drain and weigh out the required quantity.
Lotus roots, boiled	Prepare lotus root by slicing off the end of each section and then peeling it with a paring knife or vegetable peeler. You can then slice it in either very thin rounds or thicker rounds. Since cut lotus root turns brown once it comes into contact with air, once peeled, quickly rinse under cold water and after cutting up, place into cold water for 5 to 10 minutes. Bring a pot of water to the boil, boil lotus root for a couple of minutes, drain and cool under running water, drain well before weighing required amount.
Lychees, tinned	Drain and weigh out the required quantity.
Mandarins, raw	Peel and remove outer skin and separate into segments.
Mangetout, steamed	Top and tail the pods. Leave whole or slice. Place in a steamer. Steam over a pan of simmering water for 3-4 minutes or until tender.
Mango, dried	Dried, weigh out quantity required.
Mango, raw	Using a potato peeler finely peel off the skin, then hold the mango in one hand and, with a small knife, cut out a slice, taking the knife down to the stone either side of it, then remove the slice and carry on cutting slices all the way round.
Mangosteen, raw	Look for mangosteens with fresh green stems and avoid those with brown stems. Score the skin’s circumference with a sharp knife, being careful not to cut into the flesh. Peel or twist off the top and scoop out the segments.
Mooli (daikon), green, raw	Rinse and peel the mooli, chop the mooli radish finely.
Mooli, (daikon), white, raw	Rinse and peel the mooli, chop the mooli radish finely.
Mushrooms, beech (shimoji brown), lightly fried	Slicing off the base of the cluster separates them into individual stems. Give them a brief rinse to remove any dirt. Cook on medium heat for 3-5 minutes. Use same oil for all mushrooms e.g. rapeseed oil, olive oil.
Mushrooms, beech (shimoji white), lightly fried	Slicing off the base of the cluster separates them into individual stems. Give them a brief rinse to remove any dirt. Cook on medium heat for 3-5 minutes. Use same oil for all mushrooms e.g. rapeseed oil, olive oil.
Mushrooms, button, lightly fried	Give a quick rinse or wipe with kitchen towel. Slice off any hard parts of stems and slice in half. Saute in small amount of oil for 5-8 minutes. Use same oil for all mushrooms e.g. rapeseed oil, olive oil.

Appendix 1. *Continued*

Food	Instructions
Mushrooms, chestnut, lightly fried	Give a quick rinse or wipe with kitchen towel. Slice off any hard parts of stems and slice in half. Saute in small amount of oil for 5-8 minutes. Use same oil for all mushrooms e.g. rapeseed oil, olive oil.
Mushrooms, closed cap, lightly fried	Give a quick rinse or wipe with kitchen towel. Slice off any hard parts of stems and slice in half. Saute in small amount of oil for 5-8 minutes. Use same oil for all mushrooms e.g. rapeseed oil, olive oil.
Mushrooms, oyster, lightly fried	Give a quick rinse and wipe with kitchen towel. Slice off any hard parts of stems and slice in half. Saute in small amount of oil for 5-8 minutes. Use same oil for all mushrooms e.g. rapeseed oil, olive oil.
Mushrooms, portobello, lightly fried	Give a quick rinse or wipe with kitchen towel. Slice off any hard parts of stems and slice in half. Saute in small amount of oil for 5-8 minutes. Use same oil for all mushrooms e.g. rapeseed oil, olive oil.
Mushrooms, shitake, lightly fried	Give a quick rinse or wipe with kitchen towel, slice off any hard parts of stems and slice in half. Saute in small amount of oil for 5-8 minutes. Use same oil for all mushrooms e.g. rapeseed oil, olive oil.
Mustard greens, boiled	Thoroughly wash the mustard greens in 2 to 3 changes of water, or until you cannot feel any grit in the bottom of the bowl. Trim off and discard the thick stems and coarsely chop the leaves. Bring 1 cup of the water to boil in a saucepan. Add the washed greens in handfuls, waiting to add the next bunch until the leaves in the pot begin to wilt. Once all of the greens are in the pot, cover and reduce the heat to low. Simmer until the greens are tender, 15 to 20 minutes. Drain the greens in a colander, squeezing out any excess moisture.
Nectarines, raw	Remove the seed by cutting the nectarine around the seam, twist to separate the halves and remove seed, chop to required quantity.
Orange, raw	Peel orange skin keeping flesh intact.
Pak choi (bok choy), steamed	Wash, roughly chop the leaves into wider strips and the stalks into slightly smaller pieces. Place in a steamer. Steam over a pan of simmering water for 3 minutes or until just wilted.
Palm seeds, tinned	Drain and measure out the required quantity.
Papaya, raw	Cut the papaya in half lengthways. Using a teaspoon, scoop out the black seeds and discard. Use a small sharp knife to cut the flesh away from the skin of each half. Discard the skin and slice the flesh.
Parsnip, boiled	Bring a pot of water to a boil. Trim the root and leaf ends of the parsnips. Scrub the parsnips with a vegetable brush while rinsing under cool water. Peel any part of the outer parsnip that is not favourable to eat. Place the parsnips into the boiling water and reduce to a simmer. Cook for 5 - 15 minutes until the parsnips are tender.
Parsnip, roasted	Preheat oven to 220°C/200°C fan. Wash and peel parsnips and cut diagonally into 1.5 cm slices. Toss in oil and spread on single baking sheet/tin. Roast for 20 minutes, turn and roast for another 15 minutes until brown and tender.
Passion fruit, raw	Edible portion only. Cut in half and scoop out the edible portion.
Pea aubergine (pea eggplant/turkey berry), sauteed	Heat small amount of oil on pan. Reduce heat and sweat in frying pan to reduce moisture for a few minutes.
Peaches, raw	Skin and flesh only. Remove the seed by cutting the peach around the seam, twist to separate the halves and remove seed, chop to required quantity.
Peppers (capsicum), green, raw	Chop, deseed and measure out required quantity of the remainder.
Peppers (capsicum), red, raw	Chop, deseed and measure out required quantity of the remainder.
Peppers (capsicum), yellow, raw	Chop, deseed and measure out required quantity of the remainder.
Persimmon (sharon fruit), raw	Slice up and measure out required quantity.
Physalis (cape gooseberry/groundcherry)	Remove leaf. Measure out the required quantity.

Appendix 1. *Continued*

**Food**	**Instructions**
Plantain, green, boiled	Place a large pot on medium heat. Add water and bring to a boil. Wash the plantain and cut off its ends. Boil the plantain with the peels or without. Leave the peels on and slice the plantain into 2-cm circular pieces. Add the sliced plantain to the water. Boil for about 15 to 25 minutes to soften completely. Turn off the heat when the boiled plantain pieces have a vibrant yellow colour and a fork can easily go straight through the softened plantain. Drain the water out. Give the pieces a quick rinse with cold water to remove any residual white starchy froth. Remove skin after the plantain has cooled down. Measure out required amount.
Plantain, green, fried	Peel plantains by cutting off both ends and cutting a slit down the side of each plantain. Remove the plantain peel by peeling it side to side rather than lengthwise. It will come off in sections. You may need to use the knife at the edge of each section to help loosen it from the flesh. Cut the peeled plantains into 1/2 cm thick slices diagonally preferably because it provides a larger surface for caramelisation; it can be cut straight across into rounds. Drizzle just enough oil into a nonstick skillet to coat the bottom of the pan, and place it on medium heat. Add small amount of oil to the pan and when the oil begins to shimmer, but not smoke, add plantains (work in batches). Fry for 1 ½ minutes on one side, flip and cook for 1 minute on the other side.
Plantain, yellow, boiled	Place a large pot on medium heat. Add water and bring to a boil. Wash the plantain and cut off its ends. Boil the plantain with the peels or without. Leave the peels on and slice the plantain into 2-cm circular pieces (thicker slices will require a longer cook time). Add the sliced plantain to the water. Boil for about 15 to 25 minutes to soften completely. Turn off the heat when the boiled plantain pieces have a vibrant yellow colour and a fork can easily go straight through the softened plantain. Strain the water out. Give the pieces a quick rinse with cold water to remove any residual white starchy froth. Remove skin after the plantain has cooled down. Measure out required amount.
Plantain, yellow, fried	Peel plantains by cutting off both ends and cutting a slit down the side of each plantain. Remove the plantain peel by peeling it side to side rather than lengthwise. It will come off in sections. You may need to use the knife at the edge of each section to help loosen it from the flesh. Cut the peeled plantains into ½ cm thick slices diagonally preferably because it provides a larger surface for caramelisation; it can be cut straight across into rounds. Drizzle just enough oil into a nonstick skillet to coat the bottom of the pan, and place it on medium heat. Add small amount of oil to the pan and when the oil begins to shimmer, but not smoke, add plantains (work in batches). Fry for 1 ½ minutes on one side, flip and cook for 1 minute on the other side.
Pomegranate, seeds only, raw	To seed, cut the pomegranate in half, push out the centre part, hold each half over a bowl with the seeds facing down and tap the skin with a wooden spoon to release the seeds, measure out the required quantity.
Potatoes, baby, baked	Heat the oven to 220°C/200°C fan. Pierce the potato with a fork or sharp knife at least 4 times all the way around. Put on the top shelf of the oven. Bake for 20 minutes. Turn heat down to 190°C/170°C fan and bake for 45 mins to 1 hr until the skin is crisp and the flesh is soft.
Potatoes, baby, boiled	Wash (leave unpeeled) and place whole or cut potatoes in boiling water. Turn the water down to a gentle boil and cook for 10 to 20 minutes, depending on the type and the size of the potato pieces. Check frequently to avoid overcooking.

Appendix 1. *Continued*

Food	Instructions
Potatoes, baby, roasted	Put a roasting tin in the oven (one big enough to take the potatoes in a single layer) and heat oven to 200°C/fan 180°C. Drop the potatoes into a large pan and pour in enough water to barely cover them, wait for the water to boil. As soon as the water reaches a full rolling boil, lower the heat, put your timer on and simmer the potatoes uncovered, reasonably vigorously, for 2 minutes. Meanwhile put 50ml olive oil into the hot roasting tin and heat it in the oven for a few minutes until hot. Drain the potatoes in a colander, then shake the colander back and forth a few times to fluff up the outsides. Carefully put the potatoes into the hot roasting tin - they will sizzle as they go in – then turn and roll them around so they are coated all over. Spread them in a single layer making sure they have plenty of room. Roast the potatoes for 15 minutes, then take them out of the oven and turn them over. Roast for another 15 minutes and turn them over again. Put them back in the oven for another 10-20 minutes, or however long it takes to get them golden and crisp.
Potatoes, Maris Piper, airfried	Slice the potatoes lengthways into 1cm thick chips and put into a bowl of cold water for 5 minutes to soak. Preheat the air-fryer to 200°C, drain the chips and pat dry with kitchen roll, cook for 20-25 minutes, shaking occasionally (every 5 minutes), until well browned.
Potatoes, Maris Piper, baked	Heat the oven to 220°C/200°C fan. Pierce the potato with a fork or sharp knife at least 6 times all the way around. Put on the top shelf of the oven. Bake for 20 minutes. Turn heat down to 190°C/170°C fan and bake for further 45 minutes to 1 hour until the skin is crisp and the flesh soft.
Potatoes, Maris Piper, boiled	Wash and peel, place whole or cut potatoes in boiling water. Turn the water down to a gentle boil and cook for 10 to 20 minutes, depending on the type and the size of the potato pieces. Check frequently to avoid overcooking.
Potatoes, Maris Piper, mashed with butter	Wash and peel, place whole or cut potatoes in boiling water. Turn the water down to a gentle boil and cook for 10 to 20 minutes, depending on the type and the size of the potato pieces. Check frequently to avoid overcooking. Once soft in middle, add a knob of butter and mash until semi smooth.
Potatoes, Maris Piper, roasted	Put a roasting tin in the oven (one big enough to take the potatoes in a single layer) and heat oven to 200°C/fan 180°C. Peel potatoes and cut each into 4 even-sized pieces if they are medium size. Drop the potatoes into a large pan and pour in enough water to barely cover them. Add salt, then wait for the water to boil. As soon as the water reaches a full rolling boil, lower the heat, put your timer on and simmer the potatoes uncovered, reasonably vigorously, for 2 minutes. Meanwhile put 50 ml olive oil into the hot roasting tin and heat it in the oven for a few minutes until hot. Drain the potatoes in a colander, then shake the colander back and forth a few times to fluff up the outsides. Carefully put the potatoes into the hot oil – they will sizzle as they go in – then turn and roll them around so they are coated all over. Spread them in a single layer making sure they have plenty of room. Roast the potatoes for 15 minutes, then take them out of the oven and turn them over. Roast for another 15 minutes and turn them over again. Put them back in the oven for another 10-20 minutes, or however long it takes to get them golden and crisp.
Potatoes, Rooster, airfried	Slice the potatoes lengthways into 1 cm thick chips and put into a bowl of cold water for 5 minutes to soak.. Preheat the airfrier to 200°C, drain the chips and pat dry with kitchen roll, cook for 20-25 minutes, shaking occasionally (every 5 minutes), until well browned.

Appendix 1. *Continued*

Food	Instructions
Potatoes, Rooster, baked	Heat the oven to 220°C/200°C fan. Pierce the potato with fork or sharp knife at least 6 times all the way around. Put on the top shelf of the oven. Bake for 20 minutes. Turn heat down to 190C/170C fan and bake for further 45 minutes to 1 hour until the skin is crisp and the flesh soft.
Potatoes, Rooster, roasted	Put a roasting tin in the oven (one big enough to take the potatoes in a single layer) and heat oven to 200°C/fan 180°C. Peel potatoes and cut each into 4 even-sized pieces if they are medium size. Drop the potatoes into a large pan and pour in enough water to barely cover them. Add salt, then wait for the water to boil. As soon as the water reaches a full rolling boil, lower the heat, put your timer on and simmer the potatoes uncovered, reasonably vigorously, for 2 minutes. Meanwhile put 50 ml olive oil into the hot roasting tin and heat it in the oven for a few minutes until hot. Drain the potatoes in a colander, then shake the colander back and forth a few times to fluff up the outsides. Carefully put the potatoes into the hot oil – they will sizzle as they go in – then turn and roll them around so they are coated all over. Spread them in a single layer making sure they have plenty of room. Roast the potatoes for 15 minutes, then take them out of the oven and turn them over. Roast for another 15 minutes and turn them over again. Put them back in the oven for another 10-20 minutes, or however long it takes to get them golden and crisp.
Potatoes, white, boiled	Wash and peel, place whole or cut potatoes in boiling water. Turn the water down to a gentle boil and cook for 10 to 20 minutes, depending on the type and the size of the potato pieces. Check frequently to avoid overcooking.
Potatoes, white, baked	Heat the oven to 220°C/200°C fan. Pierce the potato with a fork or sharp knife at least 6 times all the way around. Put on the top shelf of the oven. Bake for 20 minutes. Turn heat down to 190°C/170°C fan and bake for further 45 minutes to 1 hour until the skin is crisp and the flesh soft.
Potatoes, white, mashed with butter	Wash and peel, place whole or cut potatoes in boiling water. Turn the water down to a gentle boil and cook for 10 to 20 minutes, depending on the type and the size of the potato pieces. Check frequently to avoid overcooking. Once soft in middle, add knob of butter and mash until semi smooth.
Potatoes, white, roasted	Put a roasting tin in the oven (one big enough to take the potatoes in a single layer) and heat oven to 200°C/fan 180°C. Peel potatoes and cut each into 4 even-sized pieces if they are medium size. Drop the potatoes into a large pan and pour in enough water to barely cover them. Add salt, then wait for the water to boil. As soon as the water reaches a full rolling boil, lower the heat, put the timer on and simmer the potatoes uncovered, reasonably vigorously, for 2 minutes. Meanwhile put 50 ml olive oil into the hot roasting tin and heat it in the oven for a few minutes until hot. Drain the potatoes in a colander, then shake the colander back and forth a few times to fluff up the outsides. Carefully put the potatoes into the hot oil – they will sizzle as they go in – then turn and roll them around so they are coated all over. Spread them in a single layer making sure they have plenty of room. Roast the potatoes for 15 minutes, then take them out of the oven and turn them over. Roast for another 15 minutes and turn them over again. Put them back in the oven for another 10-20 minutes, or however long it takes to get them golden and crisp.
Prunes, dried	Dried, weigh out quantity required.
Pumpkin (skin on), roasted	Pre-heat oven to 200°C/fan 180°C. To prepare the pumpkin, use a sharp, serrated knife to cut lid off pumpkin, use a large serving spoon to scoop out seeds and fibres. Cut flesh into wedge-shaped chunks, about 2.5 cm thick each with the skin on. Roast for about 20 minutes, until the pumpkin is softened and starting to crisp at edges.

Appendix 1. *Continued*

**Food**	**Instructions**
Quince, raw	Peel quince with vegetable peeler, cut into quarters and remove core and seeds, cut away any bruises or dark spots. Pour water in the saucepan and bring to the boil. Once boiling add quince to the water, simmer for 40-50 minutes until soft. Drain well.
Radicchio, raw	Slice the radicchio in half. Cut out the white core. Shred into thin slices. Weight out the quantity required.
Raisins, dried	Dried, weigh out quantity required.
Rambutan, tinned	Drain and weigh out the required quanity.
Redcurrants, raw	Remove stem and measure out the required quantity.
Rhubarb, champagne, boiled	Cut 500 g rhubarb into inch long pieves, put into saucepan with 2 tablespoons water. Bring to boil and then simmer for 8 minutess until rhubarb is cooked but still holds it shape.
Rocket (argula), raw	Measure out required quantity.
Runner beans, boiled	Wash in cold water, drain, top and tail, remove tough strings on side of beans with a vegetable peeler, cut diagonally, bring pan of water to the boil, cook beans for 3-4 minutes, drain and rinse under cold water.
Snakefruit, raw	Use a sharp knife to cut off the top and bottom of the fruit. Make a shallow cut along the length of the fruit and peel off the skin. Be careful when peeling the skin, as the fruit has sharp, needle-like spikes that can be prickly. Once the skin is removed, you will see three lobes of fruit inside. The yellow-white lobes of pulp has a very fine, film-like layer that can also be cleaned away, almost like peeling a boiled egg. Remove the seeds from the fruit by gently pulling them out with your fingers. The seeds are inedible. Weigh out the required quantity of the remaining flesh.
Spinach, fresh, boiled	Give quick rinse before cooking. Tip spinach into boiling water. Let it wilt (1-2 minutes), drain in colander, allow excess water to run out, press with back of spoon to assist.
Spinach, frozen, boiled	Boil from frozen, place in saucepan of boiling water, bring to boil, simmer 1-2 minutes, drain in a colander, allow excess water to run out, press with back of spoon to assist.
Starfruit (carambola), raw	Wash the star fruit. Using a sharp knife, cut very shallowly down along each of the five ridges (or "star points"), removing and discarding the brown outer edge.
Sugar snap peas, boiled	Wash in cold water, drain, top and tail. Bring pan of water to the boil, cook beans for 3-4 minutes, drain and rinse under cold water.
Sultanas, dried	Dried, weigh out quantity required.
Swede (rutabaga), boiled	Cut the root off, peel and roughly dice the swede into 2 cm cubes. Place in a pan and cover with boiling water and a good pinch of salt. Boil for 20–25 minutes or until tender.
Sweet potato, oramge, airfried	Slice the potatoes lengthways into 1 cm thick chips and put into a bowl of cold water for 5 minutes to soak. Preheat the air-fryer to 200°C, drain the chips and pat dry with kitchen roll, cook for 20-25 minutes, shaking occasionally (every 5 minutes), until well browned.
Sweet potato, orange, baked	Heat the oven to 220°C/200°C fan. Prick each potato at least 6 times and put on the top shelf of the oven. Bake for 20 minutes, then turn down the oven to 190°C/170°C fan and bake for 45 minutes to 1 hour more until the skin is crisp and the inside soft.
Sweet potato, orange, boiled	Wash and peel, place whole or cut potatoes in boiling water. Turn the water down to a gentle boil and cook for 10 to 20 minutes, depending on the type and the size of the potato pieces. Check frequently to avoid overcooking. Drain well.

Appendix 1. *Continued*

**Food**	**Instructions**
Sweet potato, orange, roasted	Heat the oven to 230°C/210°C fan. Chop the sweet potatoes into 2-3 cm cubes. Tip into a large, shallow roasting tin, drizzle with the oil and toss everything together to coat in the oil, then roast for 20 minutes. Toss again to ensure the potatoes cook evenly, and roast for another 15-20 minutes until the potatoes are lightly browned on the outside and tender when pierced with a fork or knife.
Sweet potato, purple, baked	Heat the oven to 220°C/200°C fan. Prick each potato at least 6 times and put on the top shelf of the oven. Bake for 20 minutes, then turn down the oven to 190°C/170°C fan and bake for 45 minutes to 1 hour more until the skin is crisp and the inside is soft.
Sweet potato, purple, boiled	Wash and peel, place whole or cut potatoes and add to boiling water. Turn the water down to a gentle boil and cook for 10 to 20 minutes, depending on the type and the size of the potato pieces. Check frequently to avoid overcooking. Drain well.
Sweet potato, white, baked	Heat the oven to 220°C/200°C fan. Prick each potato at least 6 times and put on the top shelf of the oven. Bake for 20 minutes, then turn down the oven to 190°C/170°C fan and bake for 45 minutes to 1 hour more until the skin is crisp and the inside is soft.
Sweet potato, white, boiled	Wash and peel, place whole or cut potatoes in boiling water. Turn the water down to a gentle boil and cook for 10 to 20 minutes, depending on the type and the size of the potato pieces. Check frequently to avoid overcooking. Drain well.
Tamarind, boiled	Add seedless tamarind in a pan and add 350-400 ml water to it. Put it on heat and bring to boil. As soon as it comes to boil, turn off heat, cover the pan with a plate and let it remain like that for 25 to 30 minutes. Drain well. Then transfer mixture to a blender and blend to a fine paste.
Taro, boiled	Wash and peel and put in large pot and cover with water to fully submerge. Bring to the boil and simmer 15-20 minutes or until the taro is soft, checking frequently to avoid overcooking. Drain well.
Tomatoes, sun dried, jarred	Drain oil, dry and measure out required quantity.
Vine leaves, boiled	Wash the vine leaves thoroughly, then put in a large saucepan, cover with water and bring to the boil. Boil for 15 minutes, then drain.
Water chestnut, tinned, drained	Drain and measure out the required quantity.
Yam, baked	Heat the oven to 190°C/170°C fan. Scrub yams thoroughly with a vegetable brush, then pat dry. Prick each yam with a fork 6 times. Bake for 40-60 minutes or until tender.
Yam, boiled	Wash and peel yams, then cut into bite size cubes. Fill a pot with just enough water to cover the yams. Bring to the boil and cook for 20-25 minutes or until tender. If you can pierce the flesh with a fork or knife, they are ready.
