# Supplementary material for: Determination of the Protein and Amino Acid Content of Fruit, Vegetables and Starchy Roots for Use in Inherited Metabolic Disorders
Source: Nutrients. 2024 Aug 23;16(17):2812. doi: 10.3390/nu16172812 (PMC11397706; doi:10.3390/nu16172812)
Supplement: Supplementary file 1 [file nutrients-16-02812-s001.zip › Table S2 The Protein content of food samples based on the typical portion sizes template.pdf]

Table S2: The protein content of food samples based on 'typical' portion sizes in grams (g)

Food	Small/child portion household measure	g	Protein (g/serving)	Average/adult portion household measure	g	Protein (g/serving)
Apricot, dried	2 apricots	11	0.3	5 apricots	26	0.8
Apricot, raw	1 whole, stone removed	37	0.2	2 whole, stoned removed	77	0.4
Artichoke, globe, boiled		20	0.4		40	0.9
Artichoke, grilled, jarred	4 pieces	27	0.5	8 pieces	53	1.1
Artichoke, jerusalem, boiled		60	0.8		135	1.8
Asparagus, boiled	2 large pieces	35	1.0	4 large pieces	75	2.0
Aubergine (eggplant), grilled**		50	0.6		150	1.7
Aubergine, Thai (Thai eggplant), sauteed	1 aubergine	40	0.5	3 aubergine	120	1.5
Aubergine, white (white eggplant), grilled	1 aubergine	30	0.7	3 aubergine	90	2.1
Avocado, Haas, raw	½ avocado	55	0.7	1 avocado	110	1.4
Avocado, raw	½ avocado	45	0.5	1 avocado	90	1.0
Baby corn, boiled	3 pieces	45	0.8	6 pieces	80	1.5
Bamboo shoots, tinned	1 tablespoon	20	0.1	2 tablespoons	40	0.3
Banana, dried	7 pieces	10	0.2	14 pieces	20	0.4
Banana, raw	Small	80	1.0	Large	120	1.6
Beetroot, boiled	½ beetroot	40	1.0	1 whole	80	2.0
Bitter gourd (bitter melon/karela), boiled	¼ gourd	30	0.4	1 gourd	114	1.3
Black kale (cavolo nero), boiled		40	1.3		80	2.6
Black radish, raw	½ radish	45	0.6	1 radish	90	1.1
Blackberries, raw	3 large/6 small blackberries	15	0.3	6 large/12 small blackberries	40	0.7
Blueberries, raw	13 blueberries	20	0.1	26 blueberries	40	0.1
Broad beans, frozen, boiled	1 tablespoon/7 beans	13	0.9	3 tablespoons/26 beans	50	3.4
Broccoli, steamed	2 florets	65	2.3	4 florets	135	4.7
Broccoli, tenderstem (broccolini), airfried	1 tenderstem	22	1.4	3 tenderstem	68	4.4
Broccoli microgreens, raw	½ tablespoon	3	0.1	2 tablespoons	8	0.2
Brussel sprouts, frozen, boiled	3 sprouts	42	1.0	5 sprouts	70	1.6
Burdock root (gobo), roasted	2 tablespoons	40	2.2	4 tablespoons	80	4.3
Butternut squash, baked	2 tablespoons	40	1.5	4 tablespoons	80	3.1
Cabbage, napa (Chinese leaf), boiled	1 tablespoon	30	0.3	3 tablespoons	90	0.9
Cabbage, red, boiled	1 tablespoon	30	0.3	3 tablespoons	90	1.0
Cabbage, savoy, boiled	1 tablespoon	30	0.2	3 tablespoons	90	0.7
Cabbage, sweetheart, boiled	1 tablespoon	25	0.2	3 tablespoons	75	0.7
Cabbage, white, boiled	1 tablespoon	20	0.2	3 tablespoons	60	0.5
Cabbage, york, boiled	1 tablespoon	20	0.2	3 tablespoons	70	0.6
Cauliflower rice, frozen, microwaved	3 tablespoons	59	0.7	160 g bag	160	1.8
Cauliflower, steamed	2 florets	80	1.4	4 florets	160	2.8
Celeriac, boiled	1 tablespoon	25	0.2	3 tablespoons	75	0.5
Chayote (chow chow), boiled	1 tablespoon	30	0.1	2 tablespoons	80	0.4
Chayote (chow chow), raw	1 tablespoon	30	0.2	3 tablespoons	90	0.7
Chicory, red, raw	1 heaped tablespoon	18	0.2	3 heaped tablespoons	47	0.6
Chicory, yellow, raw	1 heaped tablespoon	13	0.1	3 heaped tablespoons	54	0.5
Choy sum, steamed		20	0.5		60	1.4
Coconut, dessicated	1 tablespoon	6	0.4	2 tablespoons	12	0.8
Cornichons, jarred, pickled	2 cornichons	15	0.2	4 cornichons	30	0.4
Courgette (zucchini), green, lightly fried	3 slices	30	0.3	6 slices	60	0.5
Cranberries, dried	½ tablespoon	12	0.1	1 tablespoon	23	0.2
Curly kale, boiled		11	0.3		38	0.9
Currants, dried	½ tablespoon	7	0.1	1 tablespoon	15	0.3

Food	Small/child portion household measure	g	Protein (g/serving)	Average/adult portion household measure	g	Protein (g/serving)
Custard apple, raw	¼ apple	30	0.7	½ apple	63	1.5
Dates, dried	1 ½ dates	13	0.3	3 dates	26	0.5
Dates, Medjool, dried	1 ½ dates	22	0.5	3 dates	44	1.0
Dok kae flower (Karturat flower), steamed	5 pieces	10	0.2	10 pieces	25	0.6
Dragon fruit, red, raw	½ fruit	75	0.6	1 fruit	200	1.5
Dragon fruit, yellow, raw	½ fruit	54	0.5	1 fruit	105	1.1
Drumsticks, boiled	3 'finger-sized' batons	40	0.8	7 'finger-sized' batons	80	1.5
Fennel, raw	¼ piece	35	0.3	½ piece	51	0.4
Figs, dried	1 fig	17	0.5	2 figs	34	1.1
Figs, raw	1 fig	33	0.3	2 figs	65	0.7
French beans, fresh, boiled	5 beans	18	0.4	10 beans	36	0.8
French beans, frozen, boiled	5 beans	17	0.3	10 beans	32	0.6
Gherkins, jarred, pickled	1 gherkin	27	0.2	2 gherkins	52	0.5
Goji berries, dried	½ tablespoon	6	0.7	1 tablespoon	12	1.4
Jackfruit, tinned		40	0.7		80	1.5
Jackfruit, young green, tinned		45	0.2		90	0.5
Jalapeno peppers, pickled, jarred	2 slices	7	0.0	6 slices	21	0.1
Kai lan (Chinese broccoli), boiled	1 tablespoon	33	0.9	3 tablespoons	99	2.6
Kiwi, raw	½ kiwi	32	0.3	1 kiwi	65	0.6
Kohlrabi, boiled		44	0.2		97	0.5
Korean pear, raw	⅛ pear	80	0.3	¼ pear	160	0.6
Kumquats, raw	2 kumquats	28	0.4	3 kumquats	55	0.9
Leeks, sauteed		25	0.2		70	0.7
Longans, tinned	5 longans	35	0.2	9 longans	65	0.4
Lotus root, boiled	2 slices	33	0.5	6 slices	100	1.6
Lychees, tinned	4 lychees	45	0.3	8 lychees	90	0.6
Mandarins, raw	1 mandarin	90	0.6	2 mandarins	180	1.2
Mangetout, steamed		25	0.9		60	2.1
Mango, dried	2 pieces	7	0.1	4 pieces	13	0.2
Mango, raw	2 thick slices	75	0.2	3 thick slices	125	0.4
Mangosteen, raw	1 mangosteen	50	0.3	2 mangosteens	100	0.6
Mooli (daikon), green, raw		35	0.4		70	0.7
Mooli (daikon), white, raw		35	0.9		70	1.8
Mushroom, beech (shimoji brown), lightly fried	1 tablespoon	16	0.4	3 tablespoons	47	1.1
Mushrooms, beech (shimoji white), lightly fried	1 tablespoon	25	1.0	3 tablespoons	75	3.1
Mushrooms, button, lightly fried	4 mushrooms	40	1.4	8 mushrooms	80	2.8
Mushrooms, chestnut, lightly fried	2 mushrooms	40	1.1	5 mushrooms	95	2.7
Mushrooms, closed cap, lightly fried	4 mushrooms	36	1.2	9 mushrooms	80	2.8
Mushrooms, oyster, lightly fried		40	1.1		80	2.1
Mushrooms, Portobello, lightly fried	½ mushroom	36	1.0	1 mushroom	73	2.1
Mushrooms, shitake, lightly fried		25	0.9		80	2.8
Mustard greens, boiled		40	0.3		80	0.6
Nectarines, raw	½ fruit	45	0.4	1 fruit	90	0.8
Orange, raw	½ orange	100	0.8	1 orange	185	1.4
Pak choi (bok choy), steamed		25	0.3		80	1.1
Palm seeds, tinned	1 tablespoon	35	<0.1	3 tablespoons	100	<0.1
Papaya, raw		70	0.4		130	0.7
Parsnip, boiled	1 tablespoon	30	0.3	3 tablespoons	60	0.6
Parsnip, roasted	1 tablespoon	20	0.3	3 tablespoons	60	0.8

Food	Small/child portion household measure	g	Protein (g/serving)	Average/adult portion household measure	g	Protein (g/serving)
Passion fruit, raw	1 fruit	25	0.5	2 fruits	50	1.0
Pea aubergine, sauteed	1 tablespoon	13	0.3	3 tablespoons	38	1.0
Peaches, raw	1/2 fruit without stone	41	0.5	1 fruit without stone	83	0.9
Peppers (capsicum), green, raw		35	0.2		70	0.4
Peppers (capsicum), red, raw		35	0.3		70	0.5
Peppers (capsicum), yellow, raw		35	0.2		70	0.4
Persimmon (sharon fruit), raw	½ persimmon	100	0.8	1 persimmon	200	1.6
Physallis (cape gooseberry/groundcherry), raw	6 fruit	25	0.3	10 fruit	38	0.5
Plaintain, green, boiled	¼ plantain	45	0.5	½ plantain	90	1.0
Plaintain, green, fried	4 slices	40	0.6	9 slices	80	1.2
Plaintain, yellow, boiled	4 slices	80	0.9	6 slices	120	1.4
Plaintain, yellow, fried	4 slices	80	0.9	6 slices	135	1.5
Pomegranate, seeds only, raw	1 tablespoon	12	0.2	2 tablespoons	24	0.3
Potatoes, baby, baked	1 potato	36	0.8	3 potatoes	96	2.1
Potatoes, baby, boiled	1 potato	40	0.7	3 potatoes	120	2.2
Potatoes, baby, roasted	1 potato	32	0.7	3 potatoes	92	2.1
Potatoes, Maris Piper, airfried		45	1.2		140	3.6
Potatoes, Maris Piper, baked	½ potato	80	1.5	1 potato	160	3.0
Potatoes, Maris Piper, boiled	1 medium potato	80	0.9	2 medium potatoes	160	1.8
Potatoes, Maris Piper, mashed with butter	1 scoop	90	1.0	2 scoops	170	1.9
Potatoes, Maris Piper, roasted		45	0.9		120	2.3
Potatoes, Rooster, airfried		45	1.3		140	4.1
Potatoes, Rooster, baked		55	1.4		220	5.7
Potatoes, Rooster, boiled		50	0.6		160	2.0
Potatoes, Rooster, roasted	1 potato	60	1.0	2 ½ potatoes	155	2.6
Potatoes, white, baked	¼ potato	60	1.1	1 potato	220	4.2
Potatoes, white, boiled	½ potato	50	0.7	1½ potatoes	180	2.5
Potatoes, white, mashed with butter	1 scoop	75	1.0	2 ½ scoops	200	2.7
Potatoes, white, roasted	½ potato	50	1.0	1 ½ potatoes	200	4.0
Prunes, dried	2 prunes	13	0.2	4 prunes	25	0.5
Pumpkin (skin on), roasted	1 slice	70	1.5	2 slices	130	2.8
Quince, raw	¼ quince	60	0.2	½ quince	120	0.4
Raddicchio, raw	1 ½ leaves	10	0.1	2 ½ leaves	25	0.2
Raisins, dried	1 tablespoon	11	0.3	2 tablespoons	22	0.5
Rambutan, tinned	2 rambutan	30	0.2	4 rambutan	65	0.3
Redcurrants, raw	1 tablespoon	20	0.3	3 tablespoons	60	1.0
Rhubarb, champagne, boiled		40	0.4		80	0.9
Rocket (argula), raw		3	0.1		10	0.3
Runner beans, boiled		30	0.4		65	0.9
Snakefruit, raw	2 fruits	34	0.2	4 fruits	68	0.3
Spinach, fresh, boiled		30	0.9		60	1.8
Spinach, frozen, boiled	1 heaped tablespoon	22	0.6	3 heaped tablespoons	66	1.7
Starfruit (carambola), raw	1 small	55	0.5	1 medium	85	0.7
Sugar snap peas, boiled	5 peas	25	0.9	10 peas	50	1.7
Sultanas, dried	1 tablespoon	20	0.6	2 tablespoons	40	1.1
Swede (rutabaga), boiled	1 ½ tablespoons	40	0.2	3 tablespoons	80	0.5
Sweet potato, orange, airfried, batch 3		60	0.8		160	2.1
Sweet potato, orange, baked, batch 1		40	0.5		160	2.1
Sweet potato, orange, baked, batch 2		40	0.3		160	1.3
Sweet potato, orange, baked, batch 3		40	0.6		160	2.4

Food	Small/child portion household measure	g	Protein (g/serving)	Average/adult portion household measure	g	Protein (g/serving)
Sweet potato, orange, boiled, batch 1		40	0.4		80	0.8
Sweet potato, orange, boiled, batch 2		40	0.3		80	0.5
Sweet potato, orange, boiled, batch 3		40	0.5		80	0.9
Sweet potato, orange, roasted, batch 1		40	0.7		80	1.4
Sweet potato, orange, roasted, batch 2		40	0.4		80	0.8
Sweet potato, orange, roasted, batch 3		40	0.7		80	1.4
Sweet potato, purple, baked		40	1.0		80	1.9
Sweet potato, purple, boiled		40	0.5		80	1.1
Sweet potato, white, baked, batch 2		40	0.2		80	0.5
Sweet potato, white, boiled, batch 1		60	0.9		120	1.8
Sweet potato, white, boiled, batch 2		60	0.4		120	0.7
Tamarind, boiled	½ tablespoon	10	0.1	1 tablespoon	20	0.2
Taro, boiled		45	0.4		90	0.8
Tomato, sundried, jarred	1 tomato	9	0.3	2 tomatoes	18	0.6
Vine leaves, boiled		15	0.5		30	1.1
Water chestnuts, tinned	2 pieces	16	0.1	6 pieces	50	0.3
Yam, baked		40	0.6		80	1.2
Yam, boiled		40	0.3		80	0.6
