# Supplementary material for: Determination of the Protein and Amino Acid Content of Fruit, Vegetables and Starchy Roots for Use in Inherited Metabolic Disorders
Source: Nutrients. 2024 Aug 23;16(17):2812. doi: 10.3390/nu16172812 (PMC11397706; doi:10.3390/nu16172812)
Supplement: Supplementary file 1 [file nutrients-16-02812-s001.zip › Table S3 The Phe content of food samples based on the typical portion sizes template.pdf]

Table S3: The Phenylalanine (Phe) content of food samples based on 'typical' portion sizes in milligrams (mg)

Food	Small/child portion			Average/adult portion		
	household measure	g	Phe (mg/serving)	household measure	g	Phe (mg/serving)
Apricot, dried	2 apricots	11	6	5 apricots	26	14
Apricot, raw	1 whole, stone removed	37	9	2 whole, stoned removed	77	18
Artichoke, globe, boiled		20	17		40	34
Artichoke, grilled, jarred	4 pieces	27	20	8 pieces	53	40
Artichoke, jerusalem, boiled		60	36		135	81
Asparagus, boiled	2 large pieces	35	28	4 large pieces	75	60
Aubergine (eggplant), grilled**		50	19		150	56
Aubergine, Thai (Thai eggplant), sauteed	1 aubergine	40	23	3 aubergine	120	69
Aubergine, white (white eggplant), grilled	1 aubergine	30	25	3 aubergine	90	76
Avocado, Haas, raw	½ avocado	55	32	1 avocado	110	64
Avocado, raw	½ avocado	45	25	1 avocado	90	50
Baby corn, boiled	3 pieces	45	34	6 pieces	80	60
Bamboo shoots, tinned	1 tablespoon	20	8	2 tablespoons	40	16
Banana, dried	7 pieces	10	8	14 pieces	20	16
Banana, raw	Small	80	34	Large	120	51
Beetroot, boiled	½ beetroot	40	18	1 whole	80	37
Bitter gourd (bitter melon/karela), boiled	¼ gourd	30	15	1 gourd	114	59
Black kale (cavolo nero), boiled		40	53		80	106
Black radish, raw	½ radish	45	17	1 radish	90	34
Blackberries, raw	3 large/6 small blackberries	15	7	6 large/12 small blackberries	40	18
Blueberries, raw	13 blueberries	20	4	26 blueberries	40	9
Broad beans, frozen, boiled	1 tablespoon/7 beans	13	37	3 tablespoons/26 beans	50	143
Broccoli, steamed	2 florets	65	64	4 florets	135	134
Broccoli, tenderstem (broccolini), airfried	1 tenderstem	22	44	3 tenderstem	68	137
Broccoli microgreens, raw	½ tablespoon	3	3	2 tablespoons	8	7
Brussel sprouts, frozen, boiled	3 sprouts	42	33	5 sprouts	70	55
Burdock root (gobo), roasted	2 tablespoons	40	56	4 tablespoons	80	113
Butternut squash, baked	2 tablespoons	40	25	4 tablespoons	80	50
Cabbage, napa (Chinese leaf), boiled	1 tablespoon	30	11	3 tablespoons	90	34
Cabbage, red, boiled	1 tablespoon	30	11	3 tablespoons	90	33
Cabbage, savoy, boiled	1 tablespoon	30	9	3 tablespoons	90	26
Cabbage, sweetheart, boiled	1 tablespoon	25	10	3 tablespoons	75	29
Cabbage, white, boiled	1 tablespoon	20	7	3 tablespoons	60	20
Cabbage, york, boiled	1 tablespoon	20	8	3 tablespoons	70	29
Cauliflower rice, frozen, microwaved	3 tablespoons	59	32	160 g bag	160	88
Cauliflower, steamed	2 florets	80	56	4 florets	160	112
Celeriac, boiled	1 tablespoon	25	7	3 tablespoons	75	20
Chayote (chow chow), boiled	1 tablespoon	30	7	2 tablespoons	80	18
Chayote (chow chow), raw	1 tablespoon	30	8	3 tablespoons	90	24
Chicory, red, raw	1 heaped tablespoon	18	6	3 heaped tablespoons	47	16
Chicory, yellow, raw	1 heaped tablespoon	13	4	3 heaped tablespoons	54	15
Choy sum, steamed		20	16		60	49
Coconut, dessicated	1 tablespoon	6	17	2 tablespoons	12	33
Cornichons, jarred, pickled	2 cornichons	15	15	4 cornichons	30	31
Courgette (zucchini), green, lightly fried	3 slices	30	11	6 slices	60	23
Cranberries, dried	½ tablespoon	12	3	1 tablespoon	23	5
Curly kale, boiled		11	11		38	38
Currants, dried	½ tablespoon	7	4	1 tablespoon	15	9

Food	Small/child portion household measure	g	Phe (mg/serving)	Average/adult portion household measure	g	Phe (mg/serving)
Custard apple, raw	¼ apple	30	16	½ apple	63	34
Dates, dried	1 ½ dates	13	7	3 dates	26	14
Dates, Medjool, dried	1 ½ dates	22	16	3 dates	44	32
Dok kae flower (Karturat flower), steamed	5 pieces	10	10	10 pieces	25	26
Dragon fruit, red, raw	½ fruit	75	46	1 fruit	200	122
Dragon fruit, yellow, raw	½ fruit	54	26	1 fruit	105	50
Drumsticks, boiled	3 'finger-sized' batons	40	21	7 'finger-sized' batons	80	43
Fennel, raw	¼ piece	35	10	½ piece	51	15
Figs, dried	1 fig	17	21	2 figs	34	42
Figs, raw	1 fig	33	13	2 figs	65	26
French beans, fresh, boiled	5 beans	18	14	10 beans	36	27
French beans, frozen, boiled	5 beans	17	13	10 beans	32	25
Gherkins, jarred, pickled	1 gherkin	27	13	2 gherkins	52	25
Goji berries, dried	½ tablespoon	6	11	1 tablespoon	12	22
Jackfruit, tinned		40	19		80	38
Jackfruit, young green, tinned		45	15		90	30
Jalapeno peppers, pickled, jarred	2 slices	7	2	6 slices	21	6
Kai lan (Chinese broccoli), boiled	1 tablespoon	33	32	3 tablespoons	99	97
Kiwi, raw	½ kiwi	32	13	1 kiwi	65	27
Kohlrabi, boiled		44	8		97	18
Korean pear, raw	⅛ pear	80	10	¼ pear	160	20
Kumquats, raw	2 kumquats	28	14	3 kumquats	55	28
Leeks, sauteed		25	11		70	30
Longans, tinned	5 longans	35	9	9 longans	65	17
Lotus root, boiled	2 slices	33	21	6 slices	100	62
Lychees, tinned	4 lychees	45	17	8 lychees	90	33
Mandarins, raw	1 mandarin	90	21	2 mandarins	180	41
Mangetout, steamed		25	18		60	42
Mango, dried	2 pieces	7	6	4 pieces	13	11
Mango, raw	2 thick slices	75	13	3 thick slices	125	21
Mangosteen, raw	1 mangosteen	50	15	2 mangosteens	100	30
Mooli (daikon), green, raw		35	8		70	17
Mooli (daikon), white, raw		35	15		70	30
Mushroom, beech (shimoji brown), lightly fried	1 tablespoon	16	12	3 tablespoons	47	36
Mushrooms, beech (shimoji white), lightly fried	1 tablespoon	25	30	3 tablespoons	75	89
Mushrooms, button, lightly fried	4 mushrooms	40	35	8 mushrooms	80	70
Mushrooms, chestnut, lightly fried	2 mushrooms	40	31	5 mushrooms	95	75
Mushrooms, closed cap, lightly fried	4 mushrooms	36	33	9 mushrooms	80	73
Mushrooms, oyster, lightly fried		40	39		80	79
Mushrooms, Portobello, lightly fried	½ mushroom	36	28	1 mushroom	73	57
Mushrooms, shitake, lightly fried		25	25		80	81
Mustard greens, boiled		40	12		80	24
Nectarines, raw	½ fruit	45	11	1 fruit	90	21
Orange, raw	½ orange	100	21	1 orange	185	39
Pak choi (bok choy), steamed		25	11		80	36
Palm seeds, tinned	1 tablespoon	35	3	3 tablespoons	100	10
Papaya, raw		70	16		130	29
Parsnip, boiled	1 tablespoon	30	17	3 tablespoons	60	34
Parsnip, roasted	1 tablespoon	20	11	3 tablespoons	60	32

Food	Small/child portion household measure	g	Phe (mg/serving)	Average/adult portion household measure	g	Phe (mg/serving)
Passion fruit, raw	1 fruit	25	31	2 fruits	50	62
Pea aubergine, sauteed	1 tablespoon	13	12	3 tablespoons	38	36
Peaches, raw	1/2 fruit without stone	41	9	1 fruit without stone	83	18
Peppers (capsicum), green, raw		35	7		70	15
Peppers (capsicum), red, raw		35	9		70	18
Peppers (capsicum), yellow, raw		35	8		70	16
Persimmon (sharon fruit), raw	½ persimmon	100	30	1 persimmon	200	60
Physallis (cape gooseberry/groundcherry), raw	6 fruit	25	15	10 fruit	38	23
Plaintain, green, boiled	¼ plantain	45	27	½ plantain	90	53
Plaintain, green, fried	4 slices	40	24	9 slices	80	48
Plaintain, yellow, boiled	4 slices	80	49	6 slices	120	73
Plaintain, yellow, fried	4 slices	80	44	6 slices	135	74
Pomegranate, seeds only, raw	1 tablespoon	12	10	2 tablespoons	24	20
Potatoes, baby, baked	1 potato	36	35	3 potatoes	96	93
Potatoes, baby, boiled	1 potato	40	33	3 potatoes	120	98
Potatoes, baby, roasted	1 potato	32	32	3 potatoes	92	93
Potatoes, Maris Piper, airfried		45	50		140	155
Potatoes, Maris Piper, baked	½ potato	80	57	1 potato	160	115
Potatoes, Maris Piper, boiled	1 medium potato	80	41	2 medium potatoes	160	82
Potatoes, Maris Piper, mashed with butter	1 scoop	90	46	2 scoops	170	87
Potatoes, Maris Piper, roasted		45	37		120	99
Potatoes, Rooster, airfried		45	51		140	157
Potatoes, Rooster, baked		55	64		220	256
Potatoes, Rooster, boiled		50	30		160	97
Potatoes, Rooster, roasted	1 potato	60	48	2 ½ potatoes	155	125
Potatoes, white, baked	¼ potato	60	49	1 potato	220	181
Potatoes, white, boiled	½ potato	50	32	1½ potatoes	180	115
Potatoes, white, mashed with butter	1 scoop	75	47	2 ½ scoops	200	125
Potatoes, white, roasted	½ potato	50	41	1 ½ potatoes	200	163
Prunes, dried	2 prunes	13	7	4 prunes	25	14
Pumpkin (skin on), roasted	1 slice	70	57	2 slices	130	106
Quince, raw	¼ quince	60	10	½ quince	120	20
Raddicchio, raw	1 ½ leaves	10	4	2 ½ leaves	25	9
Raisins, dried	1 tablespoon	11	7	2 tablespoons	22	13
Rambutan, tinned	2 rambutan	30	9	4 rambutan	65	20
Redcurrants, raw	1 tablespoon	20	12	3 tablespoons	60	37
Rhubarb, champagne, boiled		40	14		80	28
Rocket (argula), raw		3	3		10	9
Runner beans, boiled		30	12		65	27
Snakefruit, raw	2 fruits	34	9	4 fruits	68	17
Spinach, fresh, boiled		30	37		60	74
Spinach, frozen, boiled	1 heaped tablespoon	22	30	3 heaped tablespoons	66	90
Starfruit (carambola), raw	1 small	55	14	1 medium	85	21
Sugar snap peas, boiled	5 peas	25	19	10 peas	50	38
Sultanas, dried	1 tablespoon	20	13	2 tablespoons	40	27
Swede (rutabaga), boiled	1 ½ tablespoons	40	9	3 tablespoons	80	18
Sweet potato, orange, airfried, batch 3		60	51		160	136
Sweet potato, orange, baked, batch 1		40	22		160	87
Sweet potato, orange, baked, batch 2		40	21		160	85
Sweet potato, orange, baked, batch 3		40	36		160	145

Food	Small/child portion household measure	g	Phe (mg/serving)	Average/adult portion household measure	g	Phe (mg/serving)
Sweet potato, orange, boiled, batch 1		40	18		80	37
Sweet potato, orange, boiled, batch 2		40	15		80	31
Sweet potato, orange, boiled, batch 3		40	27		80	55
Sweet potato, orange, roasted, batch 1		40	44		80	88
Sweet potato, orange, roasted, batch 2		40	18		80	36
Sweet potato, orange, roasted, batch 3		40	41		80	83
Sweet potato, purple, baked		40	51		80	103
Sweet potato, purple, boiled		40	27		80	54
Sweet potato, white, baked, batch 2		40	15		80	31
Sweet potato, white, boiled, batch 1		60	49		120	99
Sweet potato, white, boiled, batch 2		60	22		120	44
Tamarind, boiled	½ tablespoon	10	6	1 tablespoon	20	12
Taro, boiled		45	22		90	44
Tomato, sundried, jarred	1 tomato	9	11	2 tomatoes	18	22
Vine leaves, boiled		15	26		30	51
Water chestnuts, tinned	2 pieces	16	5	6 pieces	50	15
Yam, baked		40	12		80	23
Yam, boiled		40	10		80	21
