# Supplementary material for: Determination of the Protein and Amino Acid Content of Fruit, Vegetables and Starchy Roots for Use in Inherited Metabolic Disorders
Source: Nutrients. 2024 Aug 23;16(17):2812. doi: 10.3390/nu16172812 (PMC11397706; doi:10.3390/nu16172812)
Supplement: Supplementary file 1 [file nutrients-16-02812-s001.zip › Table S4 The Methionine content of food samples based on the typical portion sizes template.pdf]

Table S4: The methionine (Met) content of food samples based on 'typical' portion sizes in milligrams (mg)

Food	Small/child portion household measure	g	Met (mg/serving)	Average/adult portion household measure	g	Met (mg/serving)
Apricot, dried	2 apricots	11	2	5 apricots	26	5
Apricot, raw	1 whole, stone removed	37	2	2 whole, stoned removed	77	4
Artichoke, globe, boiled		20	8		40	16
Artichoke, grilled, jarred	4 pieces	27	8	8 pieces	53	16
Artichoke, jerusalem, boiled		60	13		135	29
Asparagus, boiled	2 large pieces	35	12	4 large pieces	75	25
Aubergine (eggplant), grilled**		50	6		150	17
Aubergine, Thai (Thai eggplant), sauteed	1 aubergine	40	8	3 aubergine	120	24
Aubergine, white (white eggplant), grilled	1 aubergine	30	9	3 aubergine	90	26
Avocado, Haas, raw	½ avocado	55	13	1 avocado	110	26
Avocado, raw	½ avocado	45	9	1 avocado	90	19
Baby corn, boiled	3 pieces	45	13	6 pieces	80	23
Bamboo shoots, tinned	1 tablespoon	20	3	2 tablespoons	40	6
Banana, dried	7 pieces	10	3	14 pieces	20	5
Banana, raw	Small	80	15	Large	120	22
Beetroot, boiled	½ beetroot	40	8	1 whole	80	16
Bitter gourd (bitter melon/karela), boiled	¼ gourd	30	5	1 gourd	114	20
Black kale (cavolo nero), boiled		40	18		80	35
Black radish, raw	½ radish	45	8	1 radish	90	16
Blackberries, raw	3 large/6 small blackberries	15	2	6 large/12 small blackberries	40	5
Blueberries, raw	13 blueberries	20	1	26 blueberries	40	3
Broad beans, frozen, boiled	1 tablespoon/7 beans	13	8	3 tablespoons/26 beans	50	30
Broccoli, steamed	2 florets	65	30	4 florets	135	62
Broccoli, tenderstem (broccolini), airfried	1 tenderstem	22	16	3 tenderstem	68	49
Broccoli microgreens, raw	½ tablespoon	3	1	2 tablespoons	8	2
Brussel sprouts, frozen, boiled	3 sprouts	42	13	5 sprouts	70	22
Burdock root (gobo), roasted	2 tablespoons	40	11	4 tablespoons	80	23
Butternut squash, baked	2 tablespoons	40	18	4 tablespoons	80	35
Cabbage, napa (Chinese leaf), boiled	1 tablespoon	30	4	3 tablespoons	90	13
Cabbage, red, boiled	1 tablespoon	30	4	3 tablespoons	90	12
Cabbage, savoy, boiled	1 tablespoon	30	3	3 tablespoons	90	10
Cabbage, sweetheart, boiled	1 tablespoon	25	5	3 tablespoons	75	14
Cabbage, white, boiled	1 tablespoon	20	2	3 tablespoons	60	7
Cabbage, york, boiled	1 tablespoon	20	3	3 tablespoons	70	12
Cauliflower rice, frozen, microwaved	3 tablespoons	59	14	160 g bag	160	38
Cauliflower, steamed	2 florets	80	25	4 florets	160	50
Celeriac, boiled	1 tablespoon	25	3	3 tablespoons	75	8
Chayote (chow chow), boiled	1 tablespoon	30	2	2 tablespoons	80	5
Chayote (chow chow), raw	1 tablespoon	30	3	3 tablespoons	90	8
Chicory, red, raw	1 heaped tablespoon	18	2	3 heaped tablespoons	47	6
Chicory, yellow, raw	1 heaped tablespoon	13	1	3 heaped tablespoons	54	6
Choy sum, steamed		20	7		60	20
Coconut, dessicated	1 tablespoon	6	7	2 tablespoons	12	14
Cornichons, jarred, pickled	2 cornichons	15	3	4 cornichons	30	6
Courgette (zucchini), green, lightly fried	3 slices	30	5	6 slices	60	10
Cranberries, dried	½ tablespoon	12	1	1 tablespoon	23	2
Curly kale, boiled		11	4		38	15

Food	Small/child portion			Average/adult portion		
	household measure	g	Met (mg/serving)	household measure	g	Met (mg/serving)
Currants, dried	½ tablespoon	7	1	1 tablespoon	15	3
Custard apple, raw	¼ apple	30	6	½ apple	63	12
Dates, dried	1 ½ dates	13	2	3 dates	26	4
Dates, Medjool, dried	1 ½ dates	22	4	3 dates	44	9
Dok kae flower (Karturat flower), steamed	5 pieces	10	5	10 pieces	25	12
Dragon fruit, red, raw	½ fruit	75	24	1 fruit	200	64
Dragon fruit, yellow, raw	½ fruit	54	14	1 fruit	105	28
Drumsticks, boiled	3 'finger-sized' batons	40	10	7 'finger-sized' batons	80	19
Fennel, raw	¼ piece	35	4	½ piece	51	5
Figs, dried	1 fig	17	3	2 figs	34	6
Figs, raw	1 fig	33	5	2 figs	65	9
French beans, fresh, boiled	5 beans	18	5	10 beans	36	10
French beans, frozen, boiled	5 beans	17	5	10 beans	32	9
Gherkins, jarred, pickled	1 gherkin	27	4	2 gherkins	52	8
Goji berries, dried	½ tablespoon	6	4	1 tablespoon	12	8
Jackfruit, tinned		40	6		80	12
Jackfruit, young green, tinned		45	5		90	10
Jalapeno peppers, pickled, jarred	2 slices	7	1	6 slices	21	2
Kai lan (Chinese broccoli), boiled	1 tablespoon	33	10	3 tablespoons	99	29
Kiwi, raw	½ kiwi	32	7	1 kiwi	65	14
Kohlrabi, boiled		44	3		97	7
Korean pear, raw	⅛ pear	80	5	¼ pear	160	9
Kumquats, raw	2 kumquats	28	4	3 kumquats	55	8
Leeks, sauteed		25	4		70	10
Longans, tinned	5 longans	35	4	9 longans	65	7
Lotus root, boiled	2 slices	33	7	6 slices	100	20
Lychees, tinned	4 lychees	45	6	8 lychees	90	13
Mandarins, raw	1 mandarin	90	6	2 mandarins	180	13
Mangetout, steamed		25	6		60	15
Mango, dried	2 pieces	7	3	4 pieces	13	6
Mango, raw	2 thick slices	75	7	3 thick slices	125	12
Mangosteen, raw	1 mangosteen	50	5	2 mangosteens	100	11
Mooli (daikon), green, raw		35	3		70	6
Mooli (daikon), white, raw		35	6		70	12
Mushroom, beech (shimoji brown), lightly fried	1 tablespoon	16	4	3 tablespoons	47	12
Mushrooms, beech (shimoji white), lightly fried	1 tablespoon	25	10	3 tablespoons	75	31
Mushrooms, button, lightly fried	4 mushrooms	40	12	8 mushrooms	80	24
Mushrooms, chestnut, lightly fried	2 mushrooms	40	12	5 mushrooms	95	28
Mushrooms, closed cap, lightly fried	4 mushrooms	36	14	9 mushrooms	80	31
Mushrooms, oyster, lightly fried		40	13		80	27
Mushrooms, Portobello, lightly fried	½ mushroom	36	12	1 mushroom	73	25
Mushrooms, shitake, lightly fried		25	9		80	27
Mustard greens, boiled		40	6		80	13
Nectarines, raw	½ fruit	45	3	1 fruit	90	6
Orange, raw	½ orange	100	10	1 orange	185	18
Pak choi (bok choy), steamed		25	3		80	10
Palm seeds, tinned	1 tablespoon	35	<2	3 tablespoons	100	<5
Papaya, raw		70	5		130	9
Parsnip, boiled	1 tablespoon	30	6	3 tablespoons	60	12

Food	Small/child portion			Average/adult portion		
	household measure	g	Met (mg/serving)	household measure	g	Met (mg/serving)
Parsnip, roasted	1 tablespoon	20	4	3 tablespoons	60	12
Passion fruit, raw	1 fruit	25	8	2 fruits	50	16
Pea aubergine, sauteed	1 tablespoon	13	4	3 tablespoons	38	13
Peaches, raw	1/2 fruit without stone	41	<2	1 fruit without stone	83	<4
Peppers (capsicum), green, raw		35	3		70	5
Peppers (capsicum), red, raw		35	4		70	8
Peppers (capsicum), yellow, raw		35	3		70	6
Persimmon (sharon fruit), raw	½ persimmon	100	11	1 persimmon	200	23
Physallis (cape gooseberry/groundcherry), raw	6 fruit	25	7	10 fruit	38	10
Plaintain, green, boiled	¼ plantain	45	10	½ plantain	90	19
Plaintain, green, fried	4 slices	40	9	9 slices	80	18
Plaintain, yellow, boiled	4 slices	80	16	6 slices	120	23
Plaintain, yellow, fried	4 slices	80	14	6 slices	135	23
Pomegranate, seeds only, raw	1 tablespoon	12	4	2 tablespoons	24	8
Potatoes, baby, baked	1 potato	36	14	3 potatoes	96	36
Potatoes, baby, boiled	1 potato	40	12	3 potatoes	120	37
Potatoes, baby, roasted	1 potato	32	12	3 potatoes	92	34
Potatoes, Maris Piper, airfried		45	26		140	81
Potatoes, Maris Piper, baked	½ potato	80	31	1 potato	160	61
Potatoes, Maris Piper, boiled	1 medium potato	80	21	2 medium potatoes	160	41
Potatoes, Maris Piper, mashed with butter	1 scoop	90	21	2 scoops	170	40
Potatoes, Maris Piper, roasted		45	17		120	46
Potatoes, Rooster, airfried		45	29		140	91
Potatoes, Rooster, baked		55	31		220	125
Potatoes, Rooster, boiled		50	16		160	50
Potatoes, Rooster, roasted	1 potato	60	22	2 ½ potatoes	155	57
Potatoes, white, baked	¼ potato	60	20	1 potato	220	75
Potatoes, white, boiled	½ potato	50	15	1½ potatoes	180	52
Potatoes, white, mashed with butter	1 scoop	75	21	2 ½ scoops	200	55
Potatoes, white, roasted	½ potato	50	20	1 ½ potatoes	200	80
Prunes, dried	2 prunes	13	2	4 prunes	25	4
Pumpkin (skin on), roasted	1 slice	70	21	2 slices	130	38
Quince, raw	¼ quince	60	5	½ quince	120	10
Raddicchio, raw	1 ½ leaves	10	2	2 ½ leaves	25	4
Raisins, dried	1 tablespoon	11	2	2 tablespoons	22	4
Rambutan, tinned	2 rambutan	30	3	4 rambutan	65	6
Redcurrants, raw	1 tablespoon	20	5	3 tablespoons	60	15
Rhubarb, champagne, boiled		40	6		80	12
Rocket (argula), raw		3	1		10	3
Runner beans, boiled		30	5		65	11
Snakefruit, raw	2 fruits	34	4	4 fruits	68	9
Spinach, fresh, boiled		30	15		60	29
Spinach, frozen, boiled	1 heaped tablespoon	22	12	3 heaped tablespoons	66	36
Starfruit (carambola), raw	1 small	55	6	1 medium	85	9
Sugar snap peas, boiled	5 peas	25	6	10 peas	50	13
Sultanas, dried	1 tablespoon	20	3	2 tablespoons	40	7
Swede (rutabaga), boiled	1 ½ tablespoons	40	3	3 tablespoons	80	6
Sweet potato, orange, airfried, batch 3		60	16		160	43
Sweet potato, orange, baked, batch 1		40	9		160	36
Sweet potato, orange, baked, batch 2		40	9		160	35

Food	Small/child portion			Average/adult portion		
	household measure	g	Met (mg/serving)	household measure	g	Met (mg/serving)
Sweet potato, orange, baked, batch 3		40	12		160	48
Sweet potato, orange, boiled, batch 1		40	6		80	13
Sweet potato, orange, boiled, batch 2		40	6		80	13
Sweet potato, orange, boiled, batch 3		40	10		80	20
Sweet potato, orange, roasted, batch 1		40	16		80	32
Sweet potato, orange, roasted, batch 2		40	7		80	14
Sweet potato, orange, roasted, batch 3		40	15		80	29
Sweet potato, purple, baked		40	15		80	31
Sweet potato, purple, boiled		40	11		80	22
Sweet potato, white, baked, batch 2		40	4		80	7
Sweet potato, white, boiled, batch 1		60	16		120	32
Sweet potato, white, boiled, batch 2		60	9		120	17
Tamarind, boiled	½ tablespoon	10	2	1 tablespoon	20	4
Taro, boiled		45	6		90	12
Tomato, sundried, jarred	1 tomato	9	2	2 tomatoes	18	5
Vine leaves, boiled		15	11		30	23
Water chestnuts, tinned	2 pieces	16	3	6 pieces	50	8
Yam, baked		40	4		80	9
Yam, boiled		40	4		80	7
