# Supplementary material for: Determination of the Protein and Amino Acid Content of Fruit, Vegetables and Starchy Roots for Use in Inherited Metabolic Disorders
Source: Nutrients. 2024 Aug 23;16(17):2812. doi: 10.3390/nu16172812 (PMC11397706; doi:10.3390/nu16172812)
Supplement: Supplementary file 1 [file nutrients-16-02812-s001.zip › Table S5 The Leucine content of food samples based on the typical portion sizes template.pdf]

Table S5: The leucine (Leu) content of food samples based on 'typical' portion sizes in milligrams (mg)

Food	Small/child portion household measure	g	Leu (mg/serving)	Average/adult portion household measure	g	Leu (mg/serving)
Apricot, dried	2 apricots	11	8	5 apricots	26	18
Apricot, raw	1 whole, stone removed	37	8	2 whole, stoned removed	77	17
Artichoke, globe, boiled		20	27		40	55
Artichoke, grilled, jarred	4 pieces	27	29	8 pieces	53	58
Artichoke, jerusalem, boiled		60	41		135	92
Asparagus, boiled	2 large pieces	35	38	4 large pieces	75	82
Aubergine (eggplant), grilled**		50	19		150	56
Aubergine, Thai (Thai eggplant), sauteed	1 aubergine	40	27	3 aubergine	120	81
Aubergine, white (white eggplant), grilled	1 aubergine	30	31	3 aubergine	90	93
Avocado, Haas, raw	½ avocado	55	46	1 avocado	110	92
Avocado, raw	½ avocado	45	33	1 avocado	90	67
Baby corn, boiled	3 pieces	45	46	6 pieces	80	82
Bamboo shoots, tinned	1 tablespoon	20	11	2 tablespoons	40	23
Banana, dried	7 pieces	10	8	14 pieces	20	17
Banana, raw	Small	80	53	Large	120	79
Beetroot, boiled	½ beetroot	40	26	1 whole	80	52
Bitter gourd (bitter melon/karela), boiled	¼ gourd	30	18	1 gourd	114	70
Black kale (cavolo nero), boiled		40	92		80	183
Black radish, raw	½ radish	45	23	1 radish	90	47
Blackberries, raw	3 large/6 small blackberries	15	9	6 large/12 small blackberries	40	24
Blueberries, raw	13 blueberries	20	4	26 blueberries	40	9
Broad beans, frozen, boiled	1 tablespoon/7 beans	13	61	3 tablespoons/26 beans	50	234
Broccoli, steamed	2 florets	65	96	4 florets	135	200
Broccoli, tenderstem (broccolini), airfried	1 tenderstem	22	55	3 tenderstem	68	171
Broccoli microgreens, raw	½ tablespoon	3	3	2 tablespoons	8	9
Brussel sprouts, frozen, boiled	3 sprouts	42	46	5 sprouts	70	76
Burdock root (gobo), roasted	2 tablespoons	40	42	4 tablespoons	80	85
Butternut squash, baked	2 tablespoons	40	25	4 tablespoons	80	49
Cabbage, napa (Chinese leaf), boiled	1 tablespoon	30	13	3 tablespoons	90	40
Cabbage, red, boiled	1 tablespoon	30	14	3 tablespoons	90	42
Cabbage, savoy, boiled	1 tablespoon	30	10	3 tablespoons	90	30
Cabbage, sweetheart, boiled	1 tablespoon	25	12	3 tablespoons	75	36
Cabbage, white, boiled	1 tablespoon	20	8	3 tablespoons	60	23
Cabbage, york, boiled	1 tablespoon	20	11	3 tablespoons	70	38
Cauliflower rice, frozen, microwaved	3 tablespoons	59	46	160 g bag	160	124
Cauliflower, steamed	2 florets	80	82	4 florets	160	165
Celeriac, boiled	1 tablespoon	25	7	3 tablespoons	75	21
Chayote (chow chow), boiled	1 tablespoon	30	6	2 tablespoons	80	17
Chayote (chow chow), raw	1 tablespoon	30	9	3 tablespoons	90	27
Chicory, red, raw	1 heaped tablespoon	18	7	3 heaped tablespoons	47	18
Chicory, yellow, raw	1 heaped tablespoon	13	4	3 heaped tablespoons	54	16
Choy sum, steamed		20	24		60	72
Coconut, dessicated	1 tablespoon	6	23	2 tablespoons	12	45
Cornichons, jarred, pickled	2 cornichons	15	19	4 cornichons	30	39
Courgette (zucchini), green, lightly fried	3 slices	30	12	6 slices	60	25
Cranberries, dried	½ tablespoon	12	3	1 tablespoon	23	5
Curly kale, boiled		11	16		38	54

Food	Small/child portion household measure	g	Leu (mg/serving)	Average/adult portion household measure	g	Leu (mg/serving)
Currants, dried	½ tablespoon	7	5	1 tablespoon	15	12
Custard apple, raw	¼ apple	30	21	½ apple	63	44
Dates, dried	1 ½ dates	13	9	3 dates	26	18
Dates, Medjool, dried	1 ½ dates	22	21	3 dates	44	41
Dok kae flower (Karturat flower), steamed	5 pieces	10	17	10 pieces	25	42
Dragon fruit, red, raw	½ fruit	75	55	1 fruit	200	147
Dragon fruit, yellow, raw	½ fruit	54	29	1 fruit	105	56
Drumsticks, boiled	3 'finger-sized' batons	40	32	7 'finger-sized' batons	80	65
Fennel, raw	¼ piece	35	11	½ piece	51	16
Figs, dried	1 fig	17	27	2 figs	34	54
Figs, raw	1 fig	33	17	2 figs	65	33
French beans, fresh, boiled	5 beans	18	19	10 beans	36	38
French beans, frozen, boiled	5 beans	17	19	10 beans	32	35
Gherkins, jarred, pickled	1 gherkin	27	15	2 gherkins	52	29
Goji berries, dried	½ tablespoon	6	19	1 tablespoon	12	38
Jackfruit, tinned		40	26		80	52
Jackfruit, young green, tinned		45	18		90	37
Jalapeno peppers, pickled, jarred	2 slices	7	2	6 slices	21	5
Kai lan (Chinese broccoli), boiled	1 tablespoon	33	35	3 tablespoons	99	105
Kiwi, raw	½ kiwi	32	17	1 kiwi	65	34
Kohlrabi, boiled		44	7		97	16
Korean pear, raw	⅛ pear	80	10	¼ pear	160	21
Kumquats, raw	2 kumquats	28	18	3 kumquats	55	35
Leeks, sauteed		25	14		70	39
Longans, tinned	5 longans	35	11	9 longans	65	21
Lotus root, boiled	2 slices	33	20	6 slices	100	62
Lychees, tinned	4 lychees	45	20	8 lychees	90	40
Mandarins, raw	1 mandarin	90	18	2 mandarins	180	36
Mangetout, steamed		25	24		60	58
Mango, dried	2 pieces	7	8	4 pieces	13	14
Mango, raw	2 thick slices	75	14	3 thick slices	125	23
Mangosteen, raw	1 mangosteen	50	19	2 mangosteens	100	38
Mooli (daikon), green, raw		35	9		70	17
Mooli (daikon), white, raw		35	20		70	39
Mushroom, beech (shimoji brown), lightly fried	1 tablespoon	16	18	3 tablespoons	47	54
Mushrooms, beech (shimoji white), lightly fried	1 tablespoon	25	46	3 tablespoons	75	139
Mushrooms, button, lightly fried	4 mushrooms	40	47	8 mushrooms	80	93
Mushrooms, chestnut, lightly fried	2 mushrooms	40	45	5 mushrooms	95	106
Mushrooms, closed cap, lightly fried	4 mushrooms	36	54	9 mushrooms	80	119
Mushrooms, oyster, lightly fried		40	58		80	116
Mushrooms, Portobello, lightly fried	½ mushroom	36	40	1 mushroom	73	82
Mushrooms, shitake, lightly fried		25	38		80	120
Mustard greens, boiled		40	15		80	30
Nectarines, raw	½ fruit	45	12	1 fruit	90	23
Orange, raw	½ orange	100	24	1 orange	185	44
Pak choi (bok choy), steamed		25	13		80	42
Palm seeds, tinned	1 tablespoon	35	<2	3 tablespoons	100	<5
Papaya, raw		70	14		130	27

Food	Small/child portion household measure	g	Leu (mg/serving)	Average/adult portion household measure	g	Leu (mg/serving)
Parsnip, boiled	1 tablespoon	30	21	3 tablespoons	60	42
Parsnip, roasted	1 tablespoon	20	13	3 tablespoons	60	40
Passion fruit, raw	1 fruit	25	27	2 fruits	50	54
Pea aubergine, sauteed	1 tablespoon	13	17	3 tablespoons	38	49
Peaches, raw	1/2 fruit without stone	41	10	1 fruit without stone	83	20
Peppers (capsicum), green, raw		35	8		70	15
Peppers (capsicum), red, raw		35	9		70	17
Peppers (capsicum), yellow, raw		35	8		70	15
Persimmon (sharon fruit), raw	½ persimmon	100	32	1 persimmon	200	63
Physallis (cape gooseberry/groundcherry), raw	6 fruit	25	20	10 fruit	38	31
Plaintain, green, boiled	¼ plantain	45	32	½ plantain	90	65
Plaintain, green, fried	4 slices	40	29	9 slices	80	58
Plaintain, yellow, boiled	4 slices	80	54	6 slices	120	81
Plaintain, yellow, fried	4 slices	80	55	6 slices	135	93
Pomegranate, seeds only, raw	1 tablespoon	12	14	2 tablespoons	24	28
Potatoes, baby, baked	1 potato	36	47	3 potatoes	96	126
Potatoes, baby, boiled	1 potato	40	42	3 potatoes	120	126
Potatoes, baby, roasted	1 potato	32	43	3 potatoes	92	123
Potatoes, Maris Piper, airfried		45	60		140	186
Potatoes, Maris Piper, baked	½ potato	80	72	1 potato	160	145
Potatoes, Maris Piper, boiled	1 medium potato	80	51	2 medium potatoes	160	101
Potatoes, Maris Piper, mashed with butter	1 scoop	90	61	2 scoops	170	116
Potatoes, Maris Piper, roasted		45	52		120	138
Potatoes, Rooster, airfried		45	68		140	210
Potatoes, Rooster, baked		55	77		220	308
Potatoes, Rooster, boiled		50	39		160	123
Potatoes, Rooster, roasted	1 potato	60	70	2 ½ potatoes	155	181
Potatoes, white, baked	¼ potato	60	54	1 potato	220	198
Potatoes, white, boiled	½ potato	50	33	1½ potatoes	180	120
Potatoes, white, mashed with butter	1 scoop	75	46	2 ½ scoops	200	124
Potatoes, white, roasted	½ potato	50	41	1 ½ potatoes	200	163
Prunes, dried	2 prunes	13	8	4 prunes	25	15
Pumpkin (skin on), roasted	1 slice	70	83	2 slices	130	154
Quince, raw	¼ quince	60	12	½ quince	120	24
Raddicchio, raw	1 ½ leaves	10	5	2 ½ leaves	25	12
Raisins, dried	1 tablespoon	11	8	2 tablespoons	22	17
Rambutan, tinned	2 rambutan	30	10	4 rambutan	65	22
Redcurrants, raw	1 tablespoon	20	15	3 tablespoons	60	46
Rhubarb, champagne, boiled		40	18		80	35
Rocket (argula), raw		3	3		10	10
Runner beans, boiled		30	17		65	38
Snakefruit, raw	2 fruits	34	12	4 fruits	68	24
Spinach, fresh, boiled		30	55		60	110
Spinach, frozen, boiled	1 heaped tablespoon	22	44	3 heaped tablespoons	66	132
Starfruit (carambola), raw	1 small	55	18	1 medium	85	27
Sugar snap peas, boiled	5 peas	25	27	10 peas	50	54
Sultanas, dried	1 tablespoon	20	17	2 tablespoons	40	34
Swede (rutabaga), boiled	1 ½ tablespoons	40	11	3 tablespoons	80	23
Sweet potato, orange, airfried, batch 3		60	47		160	126
Sweet potato, orange, baked, batch 1		40	26		160	103

Food	Small/child portion			Average/adult portion		
	household measure	g	Leu (mg/serving)	household measure	g	Leu (mg/serving)
Sweet potato, orange, baked, batch 2		40	21		160	85
Sweet potato, orange, baked, batch 3		40	35		160	140
Sweet potato, orange, boiled, batch 1		40	22		80	43
Sweet potato, orange, boiled, batch 2		40	17		80	34
Sweet potato, orange, boiled, batch 3		40	27		80	54
Sweet potato, orange, roasted, batch 1		40	42		80	85
Sweet potato, orange, roasted, batch 2		40	18		80	36
Sweet potato, orange, roasted, batch 3		40	42		80	83
Sweet potato, purple, baked		40	49		80	98
Sweet potato, purple, boiled		40	37		80	73
Sweet potato, white, baked, batch 2		40	11		80	22
Sweet potato, white, boiled, batch 1		60	54		120	107
Sweet potato, white, boiled, batch 2		60	23		120	46
Tamarind, boiled	½ tablespoon	10	6	1 tablespoon	20	13
Taro, boiled		45	28		90	55
Tomato, sundried, jarred	1 tomato	9	9	2 tomatoes	18	18
Vine leaves, boiled		15	38		30	76
Water chestnuts, tinned	2 pieces	16	7	6 pieces	50	23
Yam, baked		40	12		80	24
Yam, boiled		40	11		80	22
