# Supplementary material for: Determination of the Protein and Amino Acid Content of Fruit, Vegetables and Starchy Roots for Use in Inherited Metabolic Disorders
Source: Nutrients. 2024 Aug 23;16(17):2812. doi: 10.3390/nu16172812 (PMC11397706; doi:10.3390/nu16172812)
Supplement: Supplementary file 1 [file nutrients-16-02812-s001.zip › Table S6 The Lysine content of food samples based on the typical portion sizes template.pdf]

Table S6: The lysine (Lys) content of food samples based on 'typical' portion sizes in milligrams (mg)

Food	Small/child portion household measure	g	Lys (mg/serving)	Average/adult portion household measure	g	Lys (mg/serving)
Apricot, dried	2 apricots	11	9	5 apricots	26	20
Apricot, raw	1 whole, stone removed	37	7	2 whole, stoned removed	77	15
Artichoke, globe, boiled		20	27		40	55
Artichoke, grilled, jarred	4 pieces	27	28	8 pieces	53	54
Artichoke, jerusalem, boiled		60	56		135	126
Asparagus, boiled	2 large pieces	35	38	4 large pieces	75	81
Aubergine (eggplant), grilled**		50	23		150	70
Aubergine, Thai (Thai eggplant), sauteed	1 aubergine	40	28	3 aubergine	120	83
Aubergine, white (white eggplant), grilled	1 aubergine	30	32	3 aubergine	90	97
Avocado, Haas, raw	½ avocado	55	42	1 avocado	110	84
Avocado, raw	½ avocado	45	32	1 avocado	90	65
Baby corn, boiled	3 pieces	45	49	6 pieces	80	87
Bamboo shoots, tinned	1 tablespoon	20	11	2 tablespoons	40	22
Banana, dried	7 pieces	10	8	14 pieces	20	16
Banana, raw	Small	80	46	Large	120	69
Beetroot, boiled	½ beetroot	40	36	1 whole	80	72
Bitter gourd (bitter melon/karela), boiled	¼ gourd	30	18	1 gourd	114	69
Black kale (cavolo nero), boiled		40	77		80	154
Black radish, raw	½ radish	45	31	1 radish	90	62
Blackberries, raw	3 large/6 small blackberries	15	9	6 large/12 small blackberries	40	24
Blueberries, raw	13 blueberries	20	4	26 blueberries	40	8
Broad beans, frozen, boiled	1 tablespoon/7 beans	13	63	3 tablespoons/26 beans	50	241
Broccoli, steamed	2 florets	65	143	4 florets	135	297
Broccoli, tenderstem (broccolini), airfried	1 tenderstem	22	59	3 tenderstem	68	184
Broccoli microgreens, raw	½ tablespoon	3	3	2 tablespoons	8	9
Brussel sprouts, frozen, boiled	3 sprouts	42	65	5 sprouts	70	108
Burdock root (gobo), roasted	2 tablespoons	40	53	4 tablespoons	80	107
Butternut squash, baked	2 tablespoons	40	28	4 tablespoons	80	57
Cabbage, napa (Chinese leaf), boiled	1 tablespoon	30	13	3 tablespoons	90	38
Cabbage, red, boiled	1 tablespoon	30	19	3 tablespoons	90	56
Cabbage, savoy, boiled	1 tablespoon	30	11	3 tablespoons	90	34
Cabbage, sweetheart, boiled	1 tablespoon	25	14	3 tablespoons	75	41
Cabbage, white, boiled	1 tablespoon	20	11	3 tablespoons	60	32
Cabbage, york, boiled	1 tablespoon	20	15	3 tablespoons	70	53
Cauliflower rice, frozen, microwaved	3 tablespoons	59	63	160 g bag	160	171
Cauliflower, steamed	2 florets	80	114	4 florets	160	228
Celeriac, boiled	1 tablespoon	25	9	3 tablespoons	75	28
Chayote (chow chow), boiled	1 tablespoon	30	7	2 tablespoons	80	18
Chayote (chow chow), raw	1 tablespoon	30	10	3 tablespoons	90	30
Chicory, red, raw	1 heaped tablespoon	18	6	3 heaped tablespoons	47	15
Chicory, yellow, raw	1 heaped tablespoon	13	4	3 heaped tablespoons	54	16
Choy sum, steamed		20	23		60	69
Coconut, dessicated	1 tablespoon	6	19	2 tablespoons	12	39
Cornichons, jarred, pickled	2 cornichons	15	36	4 cornichons	30	73
Courgette (zucchini), green, lightly fried	3 slices	30	14	6 slices	60	27
Cranberries, dried	½ tablespoon	12	2	1 tablespoon	23	3
Curly kale, boiled		11	16		38	57

Food	Small/child portion			Average/adult portion		
	household measure	g	Lys (mg/serving)	household measure	g	Lys (mg/serving)
Currants, dried	½ tablespoon	7	5	1 tablespoon	15	10
Custard apple, raw	¼ apple	30	21	½ apple	63	43
Dates, dried	1 ½ dates	13	7	3 dates	26	14
Dates, Medjool, dried	1 ½ dates	22	13	3 dates	44	26
Dok kae flower (Karturat flower), steamed	5 pieces	10	17	10 pieces	25	41
Dragon fruit, red, raw	½ fruit	75	45	1 fruit	200	119
Dragon fruit, yellow, raw	½ fruit	54	22	1 fruit	105	43
Drumsticks, boiled	3 'finger-sized' batons	40	28	7 'finger-sized' batons	80	57
Fennel, raw	¼ piece	35	11	½ piece	51	16
Figs, dried	1 fig	17	19	2 figs	34	37
Figs, raw	1 fig	33	16	2 figs	65	32
French beans, fresh, boiled	5 beans	18	21	10 beans	36	43
French beans, frozen, boiled	5 beans	17	21	10 beans	32	39
Gherkins, jarred, pickled	1 gherkin	27	14	2 gherkins	52	27
Goji berries, dried	½ tablespoon	6	12	1 tablespoon	12	24
Jackfruit, tinned		40	26		80	52
Jackfruit, young green, tinned		45	18		90	36
Jalapeno peppers, pickled, jarred	2 slices	7	1	6 slices	21	4
Kai lan (Chinese broccoli), boiled	1 tablespoon	33	34	3 tablespoons	99	103
Kiwi, raw	½ kiwi	32	17	1 kiwi	65	35
Kohlrabi, boiled		44	9		97	19
Korean pear, raw	⅛ pear	80	6	¼ pear	160	12
Kumquats, raw	2 kumquats	28	19	3 kumquats	55	38
Leeks, sauteed		25	19		70	52
Longans, tinned	5 longans	35	12	9 longans	65	23
Lotus root, boiled	2 slices	33	21	6 slices	100	64
Lychees, tinned	4 lychees	45	19	8 lychees	90	39
Mandarins, raw	1 mandarin	90	22	2 mandarins	180	43
Mangetout, steamed		25	33		60	78
Mango, dried	2 pieces	7	7	4 pieces	13	13
Mango, raw	2 thick slices	75	17	3 thick slices	125	29
Mangosteen, raw	1 mangosteen	50	18	2 mangosteens	100	36
Mooli (daikon), green, raw		35	14		70	27
Mooli (daikon), white, raw		35	26		70	51
Mushroom, beech (shimoji brown), lightly fried	1 tablespoon	16	17	3 tablespoons	47	49
Mushrooms, beech (shimoji white), lightly fried	1 tablespoon	25	52	3 tablespoons	75	155
Mushrooms, button, lightly fried	4 mushrooms	40	55	8 mushrooms	80	111
Mushrooms, chestnut, lightly fried	2 mushrooms	40	44	5 mushrooms	95	103
Mushrooms, closed cap, lightly fried	4 mushrooms	36	54	9 mushrooms	80	119
Mushrooms, oyster, lightly fried		40	61		80	122
Mushrooms, Portobello, lightly fried	½ mushroom	36	39	1 mushroom	73	79
Mushrooms, shitake, lightly fried		25	37		80	120
Mustard greens, boiled		40	16		80	33
Nectarines, raw	½ fruit	45	16	1 fruit	90	32
Orange, raw	½ orange	100	31	1 orange	185	57
Pak choi (bok choy), steamed		25	18		80	56
Palm seeds, tinned	1 tablespoon	35	<2	3 tablespoons	100	<5
Papaya, raw		70	13		130	25

Food **Small/child portion** **Average/adult portion**

	household measure	g	Lys (mg/serving)	household measure	g	Lys (mg/serving)
Parsnip, boiled	1 tablespoon	30	24	3 tablespoons	60	48
Parsnip, roasted	1 tablespoon	20	15	3 tablespoons	60	45
Passion fruit, raw	1 fruit	25	25	2 fruits	50	49
Pea aubergine, sauteed	1 tablespoon	13	16	3 tablespoons	38	46
Peaches, raw	1/2 fruit without stone	41	11	1 fruit without stone	83	22
Peppers (capsicum), green, raw		35	8		70	16
Peppers (capsicum), red, raw		35	9		70	18
Peppers (capsicum), yellow, raw		35	9		70	18
Persimmon (sharon fruit), raw	½ persimmon	100	34	1 persimmon	200	68
Physallis (cape gooseberry/groundcherry), raw	6 fruit	25	17	10 fruit	38	26
Plaintain, green, boiled	¼ plantain	45	32	½ plantain	90	63
Plaintain, green, fried	4 slices	40	31	9 slices	80	61
Plaintain, yellow, boiled	4 slices	80	54	6 slices	120	82
Plaintain, yellow, fried	4 slices	80	44	6 slices	135	75
Pomegranate, seeds only, raw	1 tablespoon	12	8	2 tablespoons	24	17
Potatoes, baby, baked	1 potato	36	45	3 potatoes	96	119
Potatoes, baby, boiled	1 potato	40	45	3 potatoes	120	134
Potatoes, baby, roasted	1 potato	32	43	3 potatoes	92	124
Potatoes, Maris Piper, airfried		45	66		140	207
Potatoes, Maris Piper, baked	½ potato	80	76	1 potato	160	153
Potatoes, Maris Piper, boiled	1 medium potato	80	53	2 medium potatoes	160	107
Potatoes, Maris Piper, mashed with butter	1 scoop	90	47	2 scoops	170	90
Potatoes, Maris Piper, roasted		45	49		120	131
Potatoes, Rooster, airfried		45	76		140	236
Potatoes, Rooster, baked		55	84		220	337
Potatoes, Rooster, boiled		50	37		160	120
Potatoes, Rooster, roasted	1 potato	60	69	2 ½ potatoes	155	177
Potatoes, white, baked	¼ potato	60	56	1 potato	220	205
Potatoes, white, boiled	½ potato	50	34	1½ potatoes	180	121
Potatoes, white, mashed with butter	1 scoop	75	45	2 ½ scoops	200	121
Potatoes, white, roasted	½ potato	50	46	1 ½ potatoes	200	185
Prunes, dried	2 prunes	13	5	4 prunes	25	9
Pumpkin (skin on), roasted	1 slice	70	90	2 slices	130	167
Quince, raw	¼ quince	60	12	½ quince	120	25
Raddicchio, raw	1 ½ leaves	10	3	2 ½ leaves	25	8
Raisins, dried	1 tablespoon	11	8	2 tablespoons	22	15
Rambutan, tinned	2 rambutan	30	12	4 rambutan	65	26
Redcurrants, raw	1 tablespoon	20	17	3 tablespoons	60	50
Rhubarb, champagne, boiled		40	20		80	41
Rocket (argula), raw		3	3		10	9
Runner beans, boiled		30	18		65	40
Snakefruit, raw	2 fruits	34	11	4 fruits	68	22
Spinach, fresh, boiled		30	50		60	101
Spinach, frozen, boiled	1 heaped tablespoon	22	40	3 heaped tablespoons	66	119
Starfruit (carambola), raw	1 small	55	22	1 medium	85	34
Sugar snap peas, boiled	5 peas	25	34	10 peas	50	68
Sultanas, dried	1 tablespoon	20	15	2 tablespoons	40	30
Swede (rutabaga), boiled	1 ½ tablespoons	40	18	3 tablespoons	80	35
Sweet potato, orange, airfried, batch 3		60	42		160	111
Sweet potato, orange, baked, batch 1		40	20		160	82
**Food**	**Small/child portion**			**Average/adult portion**		

	household measure	g	Lys (mg/serving)	household measure	g	Lys (mg/serving)
Sweet potato, orange, baked, batch 2		40	20		160	81
Sweet potato, orange, baked, batch 3		40	30		160	121
Sweet potato, orange, boiled, batch 1		40	18		80	36
Sweet potato, orange, boiled, batch 2		40	18		80	36
Sweet potato, orange, boiled, batch 3		40	24		80	47
Sweet potato, orange, roasted, batch 1		40	36		80	71
Sweet potato, orange, roasted, batch 2		40	16		80	33
Sweet potato, orange, roasted, batch 3		40	36		80	72
Sweet potato, purple, baked		40	37		80	74
Sweet potato, purple, boiled		40	35		80	69
Sweet potato, white, baked, batch 2		40	10		80	20
Sweet potato, white, boiled, batch 1		60	48		120	96
Sweet potato, white, boiled, batch 2		60	22		120	44
Tamarind, boiled	½ tablespoon	10	5	1 tablespoon	20	11
Taro, boiled		45	27		90	53
Tomato, sundried, jarred	1 tomato	9	11	2 tomatoes	18	22
Vine leaves, boiled		15	36		30	72
Water chestnuts, tinned	2 pieces	16	6	6 pieces	50	19
Yam, baked		40	14		80	28
Yam, boiled		40	13		80	26
