# Supplementary material for: Determination of the Protein and Amino Acid Content of Fruit, Vegetables and Starchy Roots for Use in Inherited Metabolic Disorders
Source: Nutrients. 2024 Aug 23;16(17):2812. doi: 10.3390/nu16172812 (PMC11397706; doi:10.3390/nu16172812)
Supplement: Supplementary file 1 [file nutrients-16-02812-s001.zip › Table S7 The Phe and Tyr content of food samples based on the typical portion sizes.pdf]

Table S7: The tyrosine (Tyr) and phenylalanine (Phe) content of food samples based on 'typical' portion sizes in milligrams (mg)

Food	Small/child portion				Average/adult portion			
	household measure	g	Tyr (mg/ serving)	Phe (mg/ serving)	household measure	g	Tyr (mg/ serving)	Phe (mg/ serving)
Apricot, dried	2 apricots	11	5	6	5 apricots	26	11	14
Apricot, raw	1 whole, stone removed	37	4	9	2 whole, stoned removed	77	8	18
Artichoke, globe, boiled		20	12	17		40	25	34
Artichoke, grilled, jarred	4 pieces	27	16	20	8 pieces	53	31	40
Artichoke, jerusalem, boiled		60	22	36		135	49	81
Asparagus, boiled	2 large pieces	35	21	28	4 large pieces	75	44	60
Aubergine (eggplant), grilled**		50	12	19		150	37	56
Aubergine, Thai (Thai eggplant), sauteed	1 aubergine	40	15	23	3 aubergine	120	45	69
Aubergine, white (white eggplant), grilled	1 aubergine	30	18	25	3 aubergine	90	54	76
Avocado, Haas, raw	½ avocado	55	20	32	1 avocado	110	39	64
Avocado, raw	½ avocado	45	15	25	1 avocado	90	30	50
Baby corn, boiled	3 pieces	45	25	34	6 pieces	80	45	60
Bamboo shoots, tinned	1 tablespoon	20	7	8	2 tablespoons	40	13	16
Banana, dried	7 pieces	10	4	8	14 pieces	20	8	16
Banana, raw	Small	80	18	34	Large	120	26	51
Beetroot, boiled	½ beetroot	40	18	18	1 whole	80	36	37
Bitter gourd (bitter melon/karela), boiled	¼ gourd	30	12	15	1 gourd	114	44	59
Black kale (cavolo nero), boiled		40	43	53		80	86	106
Black radish, raw	½ radish	45	11	17	1 radish	90	23	34
Blackberries, raw	3 large/6 small blackberries	15	5	7	6 large/12 small blackberries	40	12	18
Blueberries, raw	13 blueberries	20	2	4	26 blueberries	40	5	9
Broad beans, frozen, boiled	1tablespoon /7 beans	13	24	37	3 tablespoons/ 26 beans	50	93	143
Broccoli, steamed	2 florets	65	54	64	4 florets	135	112	134
Broccoli, tenderstem (broccolini), airfried	1 tenderstem	22	37	44	3 tenderstem	68	114	137
Broccoli microgreens, raw	½ tablespoon	3	2	3	2 tablespoons	8	5	7
Brussel sprouts, frozen, boiled	3 sprouts	42	25	33	5 sprouts	70	41	55
Burdock root (gobo), roasted	2 tablespoons	40	25	56	4 tablespoons	80	50	113
Butternut squash, baked	2 tablespoons	40	26	25	4 tablespoons	80	51	50
Cabbage, napa (Chinese leaf), boiled	1 tablespoon	30	7	11	3 tablespoons	90	21	34
Cabbage, red, boiled	1 tablespoon	30	8	11	3 tablespoons	90	25	33
Cabbage, savoy, boiled	1 tablespoon	30	6	9	3 tablespoons	90	19	26
Cabbage, sweetheart, boiled	1 tablespoon	25	6	10	3 tablespoons	75	18	29
Cabbage, white, boiled	1 tablespoon	20	5	7	3 tablespoons	60	15	20
Cabbage, york, boiled	1 tablespoon	20	7	8	3 tablespoons	70	25	29

Food	Small/child portion household measure	g	Tyr (mg/ serving)	Phe (mg/ serving)	Average/adult portion household measure	g	Tyr (mg/ serving)	Phe (mg/ serving)
Cauliflower rice, frozen, microwaved	3 tablespoons	59	22	32	160 g bag	160	60	88
Cauliflower, steamed	2 florets	80	39	56	4 florets	160	78	112
Celeriac, boiled	1 tablespoon	25	5	7	3 tablespoons	75	15	20
Chayote (chow chow), boiled	1 tablespoon	30	4	7	2 tablespoons	80	12	18
Chayote (chow chow), raw	1 tablespoon	30	7	8	3 tablespoons	90	21	24
Chicory, red, raw	1 heaped tablespoon	18	4	6	3 heaped tablespoons	47	10	16
Chicory, yellow, raw	1 heaped tablespoon	13	2	4	3 heaped tablespoons	54	8	15
Choy sum, steamed		20	8	16		60	25	49
Coconut, dessicated	1 tablespoon	6	10	17	2 tablespoons	12	21	33
Cornichons, jarred, pickled	2 cornichons	15	20	15	4 cornichons	30	40	31
Courgette (zucchini), green, lightly fried	3 slices	30	7	11	6 slices	60	13	23
Cranberries, dried	½ tablespoon	12	1	3	1 tablespoon	23	2	5
Curly kale, boiled		11	9	11		38	30	38
Currants, dried	½ tablespoon	7	2	4	1 tablespoon	15	4	9
Custard apple, raw	¼ apple	30	11	16	½ apple	63	22	34
Dates, dried	1 ½ dates	13	4	7	3 dates	26	8	14
Dates, Medjool, dried	1 ½ dates	22	9	16	3 dates	44	18	32
Dok kae flower (Karturat flower), steamed	5 pieces	10	8	10	10 pieces	25	20	26
Dragon fruit, red, raw	½ fruit	75	35	46	1 fruit	200	92	122
Dragon fruit, yellow, raw	½ fruit	54	17	26	1 fruit	105	33	50
Drumsticks, boiled	3 'finger-sized' batons	40	14	21	7 'finger-sized' batons	80	27	43
Fennel, raw	¼ piece	35	7	10	½ piece	51	10	15
Figs, dried	1 fig	17	12	21	2 figs	34	24	42
Figs, raw	1 fig	33	9	13	2 figs	65	17	26
French beans, fresh, boiled	5 beans	18	11	14	10 beans	36	22	27
French beans, frozen, boiled	5 beans	17	10	13	10 beans	32	18	25
Gherkins, jarred, pickled	1 gherkin	27	8	13	2 gherkins	52	16	25
Goji berries, dried	½ tablespoon	6	8	11	1 tablespoon	12	15	22
Jackfruit, tinned		40	13	19		80	26	38
Jackfruit, young green, tinned		45	10	15		90	20	30
Jalapeno peppers, pickled, jarred	2 slices	7	1	2	6 slices	21	2	6
Kai lan (Chinese broccoli), boiled	1 tablespoon	33	17	32	3 tablespoons	99	50	97
Kiwi, raw	½ kiwi	32	10	13	1 kiwi	65	20	27
Kohlrabi, boiled		44	5	8		97	11	18
Korean pear, raw	⅛ pear	80	<2	10	¼ pear	160	<5	20
Kumquats, raw	2 kumquats	28	11	14	3 kumquats	55	21	28
Leeks, sauteed		25	7	11		70	19	30
Longans, tinned	5 longans	35	6	9	9 longans	65	11	17

Food	Small/child portion household measure	g	Tyr (mg/ serving)	Phe (mg/ serving)	Average/adult portion household measure	g	Tyr (mg/ serving)	Phe (mg/ serving)
Lotus root, boiled	2 slices	33	12	21	6 slices	100	38	62
Lychees, tinned	4 lychees	45	9	17	8 lychees	90	17	33
Mandarins, raw	1 mandarin	90	14	21	2 mandarins	180	27	41
Mangetout, steamed		25	12	18		60	29	42
Mango, dried	2 pieces	7	3	6	4 pieces	13	6	11
Mango, raw	2 thick slices	75	8	13	3 thick slices	125	13	21
Mangosteen, raw	1 mangosteen	50	8	15	2 mangosteens	100	16	30
Mooli (daikon), green, raw		35	5	8		70	10	17
Mooli (daikon), white, raw		35	8	15		70	16	30
Mushroom, beech (shimoji brown), lightly fried	1 tablespoon	16	9	12	3 tablespoons	47	27	36
Mushrooms, beech (shimoji white), lightly fried	1 tablespoon	25	22	30	3 tablespoons	75	65	89
Mushrooms, button, lightly fried	4 mushrooms	40	27	35	8 mushrooms	80	53	70
Mushrooms, chestnut, lightly fried	2 mushrooms	40	23	31	5 mushrooms	95	56	75
Mushrooms, closed cap, lightly fried	4 mushrooms	36	29	33	9 mushrooms	80	64	73
Mushrooms, oyster, lightly fried		40	24	39		80	48	79
Mushrooms, Portobello, lightly fried	½ mushroom	36	25	28	1 mushroom	73	51	57
Mushrooms, shitake, lightly fried		25	19	25		80	60	81
Mustard greens, boiled		40	7	12		80	15	24
Nectarines, raw	½ fruit	45	8	11	1 fruit	90	16	21
Orange, raw	½ orange	100	14	21	1 orange	185	26	39
Pak choi (bok choy), steamed		25	2	11		80	6	36
Palm seeds, tinned	1 tablespoon	35	<2	3	3 tablespoons	100	<5	10
Papaya, raw		70	11	16		130	21	29
Parsnip, boiled	1 tablespoon	30	11	17	3 tablespoons	60	23	34
Parsnip, roasted	1 tablespoon	20	7	11	3 tablespoons	60	21	32
Passion fruit, raw	1 fruit	25	12	31	2 fruits	50	24	62
Pea aubergine, sauteed	1 tablespoon	13	7	12	3 tablespoons	38	20	36
Peaches, raw	1/2 fruit without stone	41	10	9	1 fruit without stone	83	19	18
Peppers (capsicum), green, raw		35	6	7		70	11	15
Peppers (capsicum), red, raw		35	6	9		70	12	18
Peppers (capsicum), yellow, raw		35	7	8		70	13	16
Persimmon (sharon fruit), raw	½ persimmon	100	15	30	1 persimmon	200	29	60
Physallis (cape gooseberry/groundcherry), raw	6 fruit	25	11	15	10 fruit	38	16	23
Plaintain, green, boiled	¼ plantain	45	16	27	½ plantain	90	31	53
Plaintain, green, fried	4 slices	40	13	24	9 slices	80	27	48
Plaintain, yellow, boiled	4 slices	80	25	49	6 slices	120	38	73

Food	Small/child portion household measure	g	Tyr (mg/ serving)	Phe (mg/ serving)	Average/adult portion household measure	g	Tyr (mg/ serving)	Phe (mg/ serving)
Plaintain, yellow, fried	4 slices	80	23	44	6 slices	135	39	74
Pomegranate, seeds only, raw	1 tablespoon	12	8	10	2 tablespoons	24	15	20
Potatoes, baby, baked	1 potato	36	28	35	3 potatoes	96	75	93
Potatoes, baby, boiled	1 potato	40	28	33	3 potatoes	120	84	98
Potatoes, baby, roasted	1 potato	32	27	32	3 potatoes	92	79	93
Potatoes, Maris Piper, airfried		45	40	50		140	126	155
Potatoes, Maris Piper, baked	½ potato	80	47	57	1 potato	160	95	115
Potatoes, Maris Piper, boiled	1 medium potato	80	30	41	2 medium potatoes	160	60	82
Potatoes, Maris Piper, mashed with butter	1 scoop	90	36	46	2 scoops	170	69	87
Potatoes, Maris Piper, roasted		45	27	37		120	73	99
Potatoes, Rooster, airfried		45	46	51		140	142	157
Potatoes, Rooster, baked		55	60	64		220	241	256
Potatoes, Rooster, boiled		50	28	30		160	88	97
Potatoes, Rooster, roasted	1 potato	60	40	48	2 ½ potatoes	155	104	125
Potatoes, white, baked	¼ potato	60	35	49	1 potato	220	130	181
Potatoes, white, boiled	½ potato	50	22	32	1½ potatoes	180	78	115
Potatoes, white, mashed with butter	1 scoop	75	34	47	2 ½ scoops	200	92	125
Potatoes, white, roasted	½ potato	50	34	41	1 ½ potatoes	200	136	163
Prunes, dried	2 prunes	13	3	7	4 prunes	25	6	14
Pumpkin (skin on), roasted	1 slice	70	48	57	2 slices	130	89	106
Quince, raw	¼ quince	60	6	10	½ quince	120	11	20
Raddicchio, raw	1 ½ leaves	10	2	4	2 ½ leaves	25	6	9
Raisins, dried	1 tablespoon	11	3	7	2 tablespoons	22	7	13
Rambutan, tinned	2 rambutan	30	6	9	4 rambutan	65	14	20
Redcurrants, raw	1 tablespoon	20	6	12	3 tablespoons	60	17	37
Rhubarb, champagne, boiled		40	11	14		80	23	28
Rocket (argula), raw		3	2	3		10	5	9
Runner beans, boiled		30	9	12		65	19	27
Snakefruit, raw	2 fruits	34	5	9	4 fruits	68	10	17
Spinach, fresh, boiled		30	33	37		60	66	74
Spinach, frozen, boiled	1 heaped tablespoon	22	23	30	3 heaped tablespoons	66	70	90
Starfruit (carambola), raw	1 small	55	14	14	1 medium	85	22	21
Sugar snap peas, boiled	5 peas	25	14	19	10 peas	50	28	38
Sultanas, dried	1 tablespoon	20	7	13	2 tablespoons	40	13	27
Swede (rutabaga), boiled	1 ½ tablespoons	40	6	9	3 tablespoons	80	13	18
Sweet potato, orange, airfried, batch 3		60	24	51		160	63	136
Sweet potato, orange, baked, batch 1		40	14	22		160	55	87
Sweet potato, orange, baked, batch 2		40	11	21		160	45	85
Sweet potato, orange, baked, batch 3		40	20	36		160	78	145

Food	Small/child portion household measure	g	Tyr (mg/ serving)	Phe (mg/ serving)	Average/adult portion household measure	g	Tyr (mg/ serving)	Phe (mg/ serving)
Sweet potato, orange, boiled, batch 1		40	11	18		80	23	37
Sweet potato, orange, boiled, batch 2		40	9	15		80	18	31
Sweet potato, orange, boiled, batch 3		40	16	27		80	33	55
Sweet potato, orange, roasted, batch 1		40	30	44		80	59	88
Sweet potato, orange, roasted, batch 2		40	9	18		80	19	36
Sweet potato, orange, roasted, batch 3		40	24	41		80	47	83
Sweet potato, purple, baked		40	30	51		80	59	103
Sweet potato, purple, boiled		40	18	27		80	36	54
Sweet potato, white, baked, batch 2		40	6	15		80	12	31
Sweet potato, white, boiled, batch 1		60	28	49		120	56	99
Sweet potato, white, boiled, batch 2		60	15	22		120	29	44
Tamarind, boiled	½ tablespoon	10	3	6	1 tablespoon	20	6	12
Taro, boiled		45	17	22		90	34	44
Tomato, sundried, jarred	1 tomato	9	6	11	2 tomatoes	18	12	22
Vine leaves, boiled		15	19	26		30	38	51
Water chestnuts, tinned	2 pieces	16	4	5	6 pieces	50	13	15
Yam, baked		40	10	12		80	21	23
Yam, boiled		40	6	10		80	13	21
