# Supplementary figures and images for: Determination of the Protein and Amino Acid Content of Fruit, Vegetables and Starchy Roots for Use in Inherited Metabolic Disorders
Source: Nutrients. 2024 Aug 23;16(17):2812. doi: 10.3390/nu16172812 (PMC11397706; doi:10.3390/nu16172812)

Cruciferous vegetables

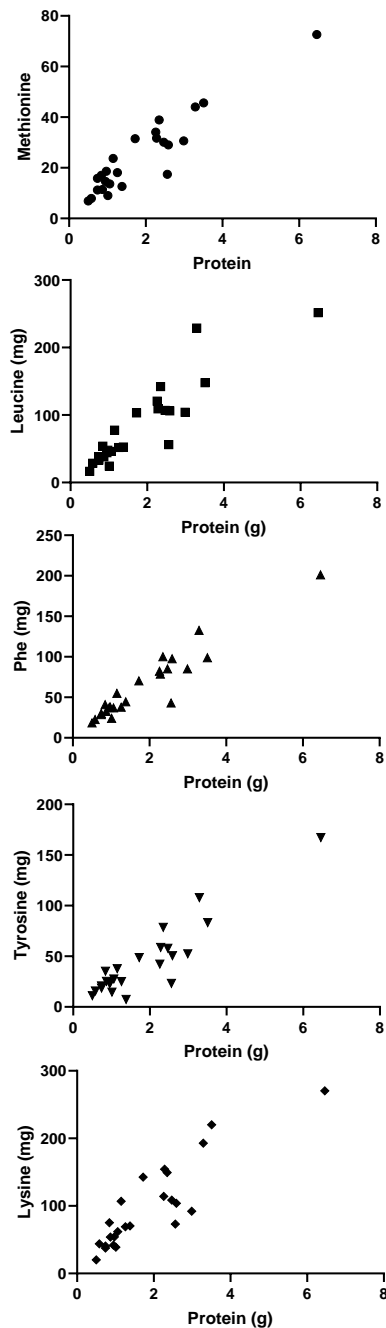

Fruit

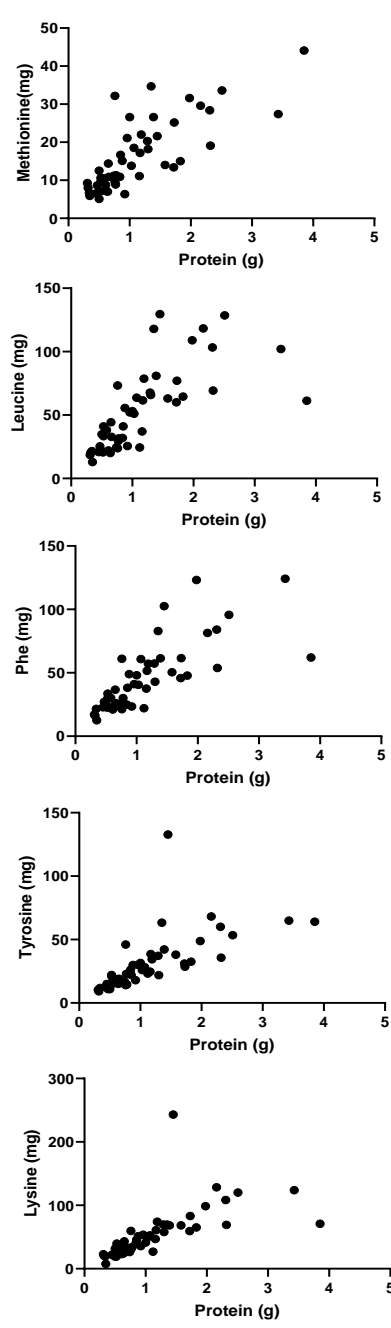

Dried Fruit

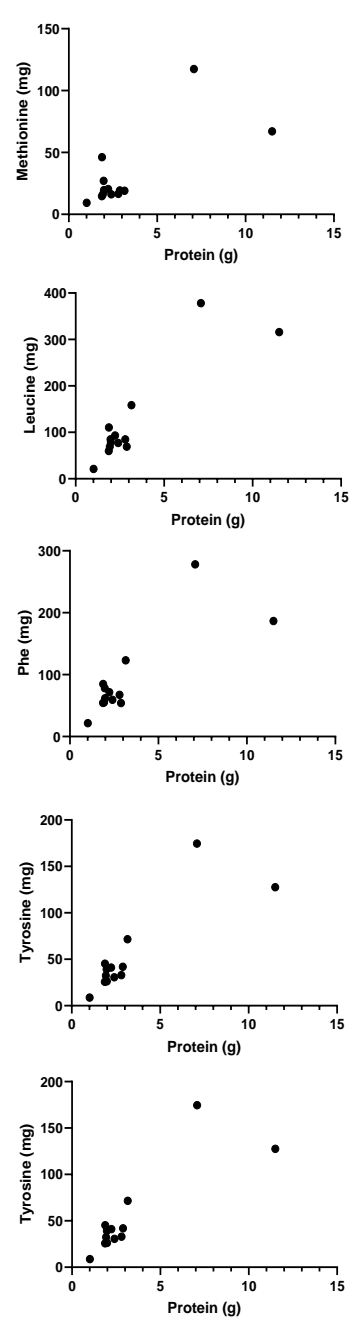

Legumes

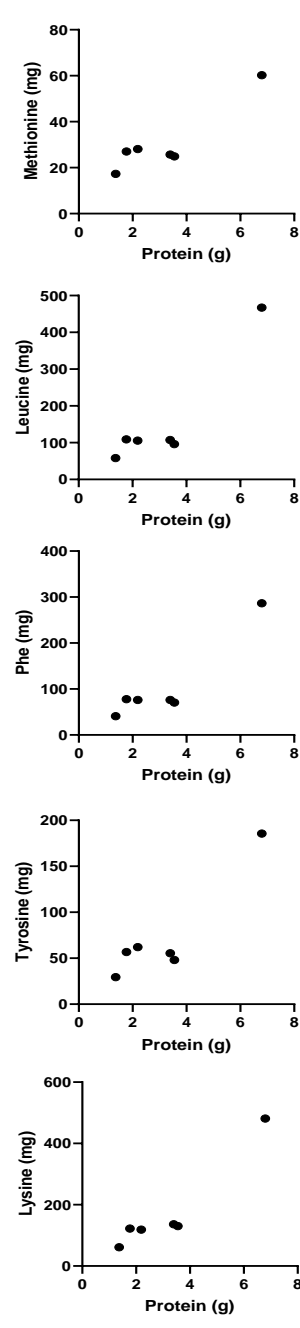

Other vegetables

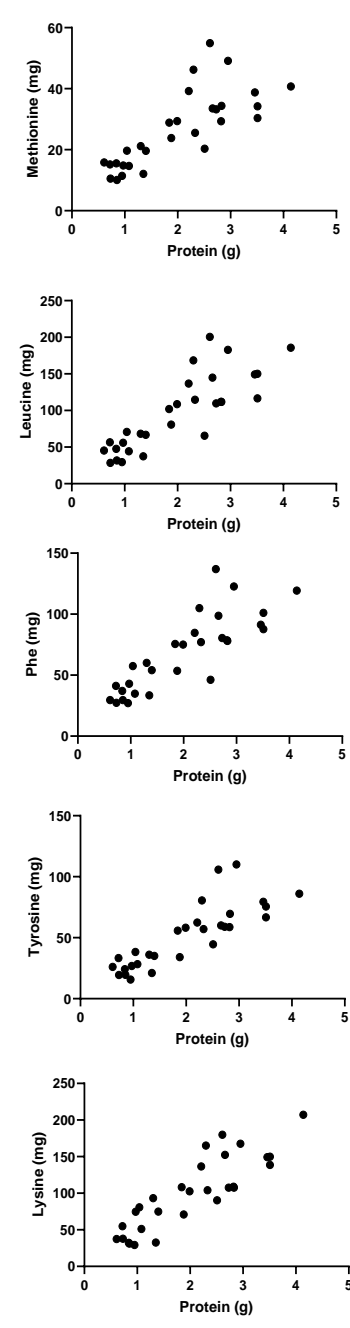

Starchy roots

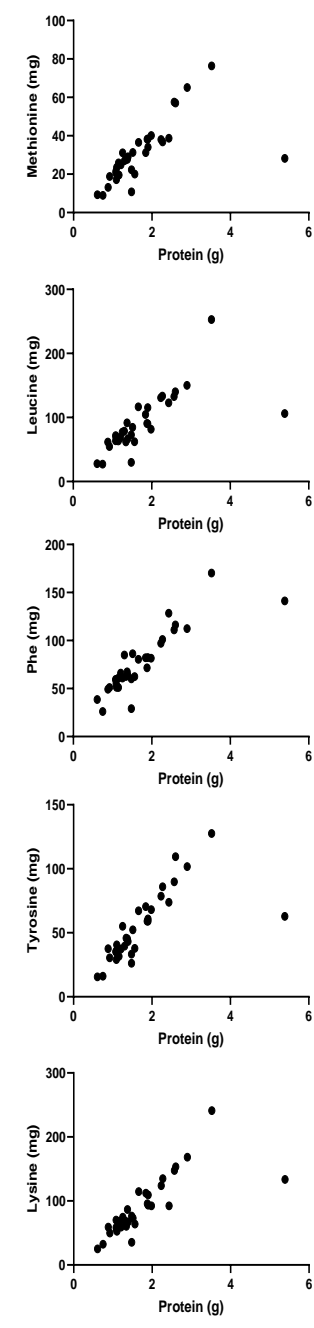

Supplement: Supplementary file 1 [file nutrients-16-02812-s001.zip › Figure S1 Correlation of protein and individual amino acids 2.pdf]
